# Non-malarial febrile illness: a systematic review of published aetiological studies and case reports from China, 1980–2015

**DOI:** 10.1186/s12879-024-09542-3

**Published:** 2024-08-20

**Authors:** Dennis K. M. Ip, Yvonne Y. Ng, Yat H. Tam, Nigel V. Thomas, Prabin Dahal, Kasia Stepniewska, Paul N. Newton, Philippe J. Guérin, Heidi Hopkins

**Affiliations:** 1 WHO Collaborating Centre for Infectious Disease Epidemiology and Control, School of Public Health, Li Ka Shing Faculty of Medicine, The University of Hong Kong, Special Administrative Region, Hong Kong, China; 2 Infectious Diseases Data Observatory (IDDO), University of Oxford, NDMRB, Old Road Campus, Oxford, OX3 7FZ UK; 3 Centre for Tropical Medicine and Global Health, Nuffield Department of Clinical Medicine, University of Oxford, Oxford, UK; 4 Lao-Oxford-Mahosot Hospital-Wellcome Research Unit, Mahosot Hospital, Vientiane, Laos; 5 London School of Hygiene and Tropical Medicine, London, WC1E 7HT UK

**Keywords:** Malaria, Non-malarial febrile illness, Aetiology

## Abstract

**Background:**

Rapid point-of-care tests for malaria are now widely used in many countries to guide the initial clinical management of patients presenting with febrile illness. With China having recently achieved malaria elimination, better understanding regarding the identity and distribution of major non-malarial causes of febrile illnesses is of particular importance to inform evidence-based empirical treatment policy.

**Methods:**

A systematic review of published literature was undertaken to characterise the spectrum of pathogens causing non-malaria febrile illness in China (1980–2015). Literature searches were conducted in English and Chinese languages in six databases: Ovid MEDLINE, Global Health, EMBASE, Web of Science™ – Chinese Science Citation Database ^SM^, The China National Knowledge Infrastructure (CNKI), and WanFang Med Online. Selection criteria included reporting on an infection or infections with a confirmed diagnosis, defined as pathogens detected in or cultured from samples from normally sterile sites, or serological evidence of current or past infection. The number of published articles, reporting a given pathogen were presented, rather than incidence or prevalence of infection.

**Results:**

A total of 57,181 records from 13 provinces of China where malaria used to be endemic were screened, of which 392 met selection criteria and were included in this review. The review includes 60 (15.3%) records published from 1980 to 2000, 211 (53.8%) from 2001 to 2010 and 121 (30.9%) from 2011 to 2015;. Of the 392 records, 166 (42.3%) were from the eastern region of China, 120 (30.6%) were from the south-west, 102 (26.0%) from south-central, and four (1.0%) were multi-regional studies. Bacterial infections were reported in 154 (39.3%) records, viral infections in 219 (55.9%), parasitic infections in four (1.0%), fungal infections in one (0.3%), and 14 (3.6%) publications reported more than one pathogen group. Participants of all ages were included in 136 (34.7%) studies, only adults in 75 (19.1%), only children in 17 (4.3%), only neonates in two (0.5%) and the age distribution was not specified in 162 (41.3%) records. The most commonly reported bacterial pathogens included Typhoidal *Salmonella* (*n* = 30), *Orientia/ Rickettsia tsutsugamushi* (*n* = 31), *Coxiella burnetii* (*n* = 17), *Leptospira* spp. (*n* = 15) and *Brucella* spp. (*n* = 15). The most commonly reported viral pathogens included Hantavirus/Hantaan virus (*n* = 89), dengue virus (DENV) (*n* = 76 including those with unknown serovars), Japanese encephalitis virus (*n* = 21), and measles virus (*n* = 15). The relative lack of data in the western region of the country, as well as in in neonates and children, represented major gaps in the understanding of the aetiology of fever in China.

**Conclusions:**

This review presents a landscape of non-malaria pathogens causing febrile illness in China over 36 years as the country progressed toward malaria elimination. These findings can inform guidelines for clinical management of fever cases and infection surveillance and prevention, and highlight the need to standardize operational and reporting protocols for better understanding of fever aetiology in the country.

**Supplementary Information:**

The online version contains supplementary material available at 10.1186/s12879-024-09542-3.

## Background

Historically, malaria had a widespread distribution with endemicity in many provinces in China, with an estimated annual incidence of thirty million cases in the 1940s [1]. Aiming to achieve malaria elimination in the country by 2020, the National Malaria Elimination Programme (NMEP) was launched in 2010 [2]. In particular, the programme’s “1-3-7” strategy set stringent standards for handling malaria cases, with a goal of reporting cases within one day, confirming and investigating within three days, and instituting appropriate public health responses to prevent further transmission within seven days. Since the NMEP launch, there has been a drastic change in the epidemiology of malaria in China, with a continuing shrinkage in the number of counties reporting malaria transmission within the previous three years. Over the last decade, autochthonous cases were reported only in more than 10 of the 2,858 mainland counties, and for 2017 and 2018, zero indigenous cases were reported in the whole country [3]. Certification of malaria elimination by the World Health Organization (WHO) was achieved by China in 2020 [4]. Prior to elimination, new cases reported in recent years mainly involved high-risk Chinese nationals, including inhabitants of border areas with other endemic South-East Asian countries, or imported infections in Chinese travellers returning from various malaria-endemic African and South-East Asian countries [3]. *Plasmodium vivax* represented the dominant malaria species of cases imported from south-east Asia (78%), while *Plasmodium falciparum* dominated in those from Africa (80%) [5].

Hence, better understanding of the major non-malarial causes of febrile illnesses in China is becoming increasingly important. The management of non-malarial febrile illness (NMFI), as suggested by a negative malaria test result, poses an increasing conundrum to clinicians and health workers in many countries, including previously malaria-endemic areas of China, because of the general lack of information regarding potential aetiological agents that need to be considered. An updated understanding regarding the identity and mapping of the distribution of NMFI-causing pathogens over the whole country would help to inform algorithms for targeted investigation and evidence-based empirical treatment on an individual patient level, and to avoid unnecessary antibiotic prescriptions and potential pressure toward antimicrobial resistance at the community level [6, 7].

Similar NMFI mapping efforts have recently been published for sub-Saharan Africa [8], Latin America [9], and South and Southeast Asia [10]. Although these studies have contributed to an improved baseline understanding of the different bacterial, viral, and parasitic pathogens for NMFIs across the three continents/regions, the situation in China remains a major knowledge gap in the global picture of NMFI. Here we report the results from a large systematic review of the published literature on records of non-malaria pathogens that may cause febrile illness in China, from 1980 to 2015. This review aims to provide a more comprehensive assessment of the nature and geographical distribution of infections that may cause febrile illness in China over the past few decades, so as to inform clinical management guidelines, and to identify gaps in knowledge for a future research agenda in relation to needed policy on the surveillance, control and prevention of febrile illnesses in China.

## Methods

### Literature search

This systematic review followed the Preferred Reporting Items for Systematic reviews and Meta-Analyses (PRISMA) guidelines [11] and searched published articles describing NMFI in China in three English databases (Ovid MEDLINE, Global Health and Embase) and three Chinese databases (Web of Science ^TM^ – Chinese Science Citation Database ^SM^, The China National Knowledge Infrastructure (CNKI), WanFang Med Online). Search terms included keywords specific for pathogens and symptoms, combined with “China” (Supplemental file S1). The search was restricted temporally to articles published over a period of 36 years, from 1980 to 2015 inclusive, and geographically to provinces/provincial level administrations documented to have known malaria transmission according to the WHO [12] and the United States Centers for Disease Control and Prevention (US CDC) [13], or with malaria outbreak(s) reported since 1990 [14]. These included 12 out of the 31 provinces in Mainland China, namely Anhui, Chongqing, Fujian, Guangxi, Guizhou, Hainan, Henan, Hubei, Jiangsu, Tibet, Yunnan and Zhejiang, covering 41.8% of the total population as of the 2020 Population Census of China [15]. The search was not limited by study design or patient age. Clinical criteria were not included; the review aimed to identify pathogen presence rather than clinical evidence of infection. This review was conducted according to a protocol previously registered with the international prospective register of systematic reviews (PROSPERO Registration ID: CRD42016049281).

### Eligibility and study selection

Titles and abstracts were first screened for compliance with the selection criteria. After selection, full papers were reviewed. The criteria used for inclusion and exclusion of the studies are provided in Table 1. One author independently applied the criteria to identify studies qualified for the review (YYN), and two other authors independently verified the results (DKMI and YHT) with discrepancies settled in consensus.

**Table 1:** Inclusion and exclusion criteria

Inclusion criteria	Reporting on pathogens causing fever in human (inpatients or outpatients)
	Studies conducted in the targeted geographical areas
	Abstract and full text available in English or Chinese
	Samples tested from normally sterile sites^1^
	Samples analysed in a laboratory setting
	Total number of individuals tested is clearly stated for population-based studies (case reports and case series were categorised separately and did not need to meet this criterion)
**Exclusion criteria**	Published before 1980
	Primary focus on malaria, HIV, or tuberculosis
	Non-clinical studies (descriptions of laboratory methods, modelling studies, economic evaluations, opinion pieces)
	Drug or vaccine trial
	Studies conducted in travellers
	Other studies of disease not including laboratory identification of pathogens causing fever

^1^The definition of a confirmed diagnosis was restricted to pathogens detected in or cultured from samples from normally sterile sites (e.g. bacterial or fungal isolates cultured from the blood, cerebrospinal fluid, arthrocentesis or paracentesis fluid, etc., or virus or parasite detection in the blood or cerebrospinal fluid) or serological evidence of current or past infection

### Data extraction

Data on pre-defined variables were extracted from the selected articles and captured in an online database hosted by the Infectious Diseases Data Observatory (IDDO) at the University of Oxford [16]. Data extracted included author details, year of publication, start and end year of the study, geo-coded study site location, study design, participants’ age-range, specimen type/s, laboratory method/s employed, number of participants tested for each fever-causing pathogen, and number of confirmed cases for each infection. Frequency was recorded as zero when a specific pathogen was tested for but not detected, and no result was recorded if testing for a specific pathogen was not done. The numbers of different aetiologies identified for the pathogens in each study were recorded as the main outcome measure.

### Case definitions

We searched for reports of human infection with non-malarial pathogens known to cause febrile illness, with the diagnosis confirmed by laboratory identification of the relevant pathogens detected in or cultured from samples from normally sterile sites (e.g. blood, cerebrospinal fluid, arthrocentesis or paracentesis fluid, etc.), or by serological evidence of current or past infection. Clinical criteria were not included.

### Study type

Three types of studies were included. “Case series” included reports describing individual cases or series of patients with the same specific infection. In “fever series”, groups of febrile patients were tested for different causative agents, with the number of individuals tested positive reported as the numerator, and the number of population tested reported as the denominator. In “seroprevalence studies”, serum samples collected from symptomatic and/or asymptomatic people during a specific time point were tested for different targeted pathogens.

The age ranges of patients were categorized as: neonates (< 28 days of life), infants (1–12 months), children (1–12 years), and older children and adults (13 + years). Some studies did not report age details of participants, or reported aggregated data for all age groups without stratifying by age.

### Categorisation of infections

Infections were categorised according to the pathogenic organism, as bacterial, viral, fungal, or parasitic. Sub-categories based on principal mode of transmission (airborne, food and/or water-borne, vector-borne, and contact-based [direct, indirect, droplet or droplet nuclei]) were also defined. Infections caused by all serotypes of Salmonella except for Typhi, Paratyphi A, Paratyphi B, and Paratyphi C were defined as non-typhoidal Salmonella (NTS). Details regarding the categorisation of the infections are presented in Supplemental file S3.

### Data visualization by online interactive map

The reported location of each study site was geo-coded onto an on-line interactive map (surveyor) hosted by IDDO [16]. The software for visualization is open source and available on GitHub (https://github.com/WorldwideAntimalarialResistanceNetwork/WWARN-Maps-Surveyor). The map displays the included studies across the different regions of China. Details including study title, authors, year, study site and province are revealed on clicking the geocoded marker on the interactive map, with studies searchable by country, pathogen, year, and patient age group.

### Statistical analysis

Each “published article” is adopted as the unit of analysis. Descriptive statistics were presented: frequencies and percentages for categorical variables. Meta-analysis was not possible due to heterogeneity in study design, pathogens examined, and the laboratory and reporting practices. Estimation of overall pathogen prevalence was precluded by the varied ascertainment practices and due to the lack of suitable population level denominator. All analyses were carried out using R software version 4.0.4 (R Foundation for Statistical Computing, Vienna, Austria), and graphs were generated using ggplot2 library [17].

### Risk of bias assessment

While a standard “risk of bias” assessment based on the evaluation of selection, performance, attrition, and reporting is not applicable for this review, the risk of bias regarding the different study designs are discussed. [18, 19]. We assessed quality of the studies included, based on study design and laboratory methods used for identification of the pathogens. Case reports/series were considered to be at a particular high risk of bias because of their tendency to report atypical presentations and cases detected in epidemiological outbreaks. Seroprevalence studies, with their frequent inability to infer acute or past infection, risk inaccurate differentiation of recovered individuals from those with acute infections. Fever series, on the other hand, were considered to be at a lower risk of bias if individuals were tested for a number of causative agents at the same time with clear reporting of the total number of participants being tested. Studies that did not report the diagnostic methods used for pathogen detection were also considered to be at high risk of bias. The assessment of risk of bias in studies included is presented in Supplemental file S3.

## Results

### Search results

**Fig. 1:**
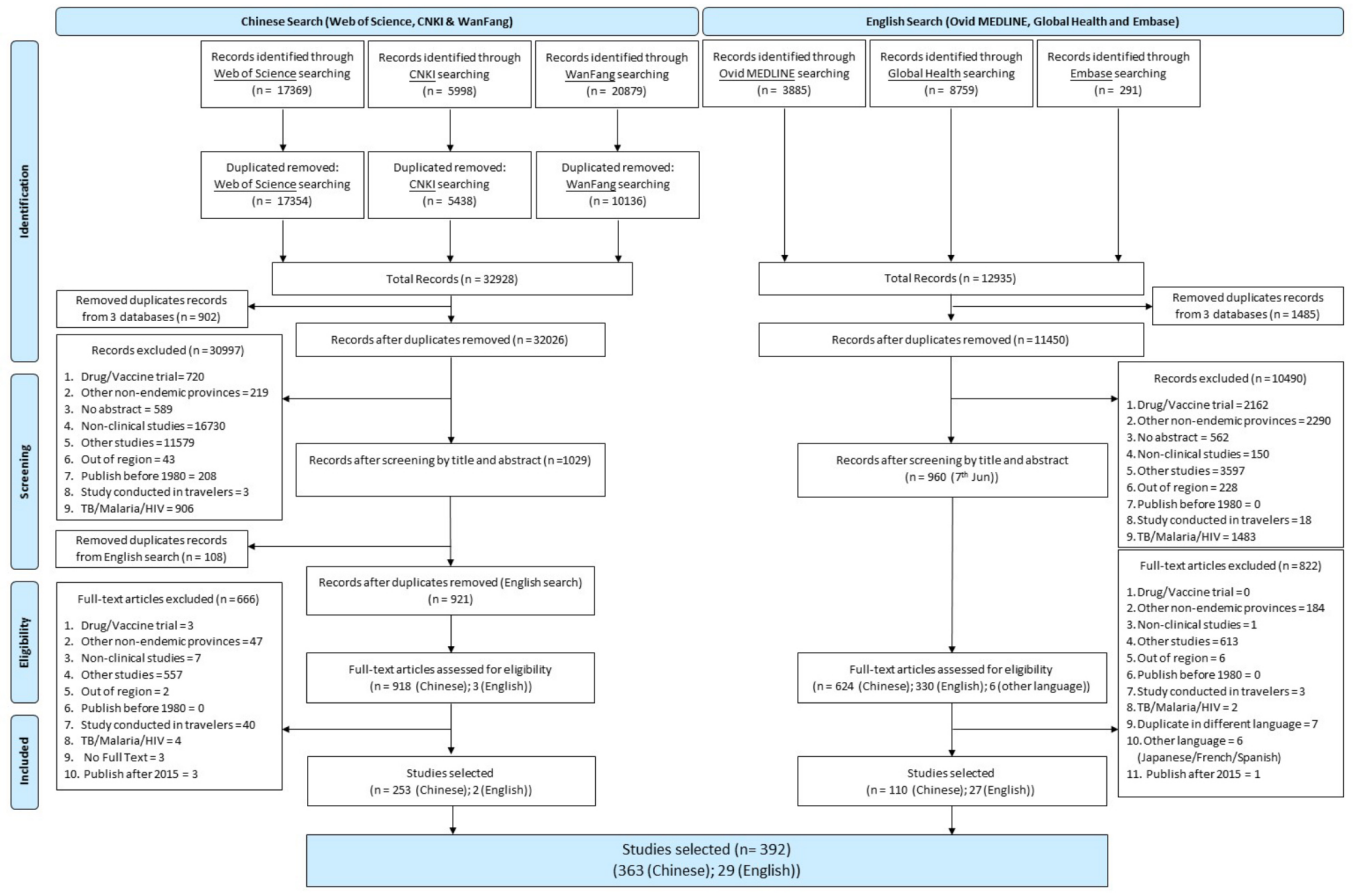
Preferred Reporting Items for Systematic Reviews and Meta-Analyses (PRISMA) flow diagram of publications screened in a systematic review of published aetiological studies and case reports from China, 1980–2015

The database search identified a total of 57,181 articles (44,246 from Chinese and 12,935 from English language databases). After excluding 13,705 duplicated articles and a further 41,953 articles that did not meet the inclusion criteria during the screening, 392 articles were included in this review (Fig. 1). These studies were published from 1980 to 2015, inclusive. 93% (*n* = 363) were published in Chinese and 7.3% (*n* = 29) were in English. The majority (84.7%) of the reviewed papers, especially those reporting bacterial and viral pathogens, were published after the year 2000 (Supplemental file S2).

### Spatio-temporal distribution of included papers

**Fig. 2:**
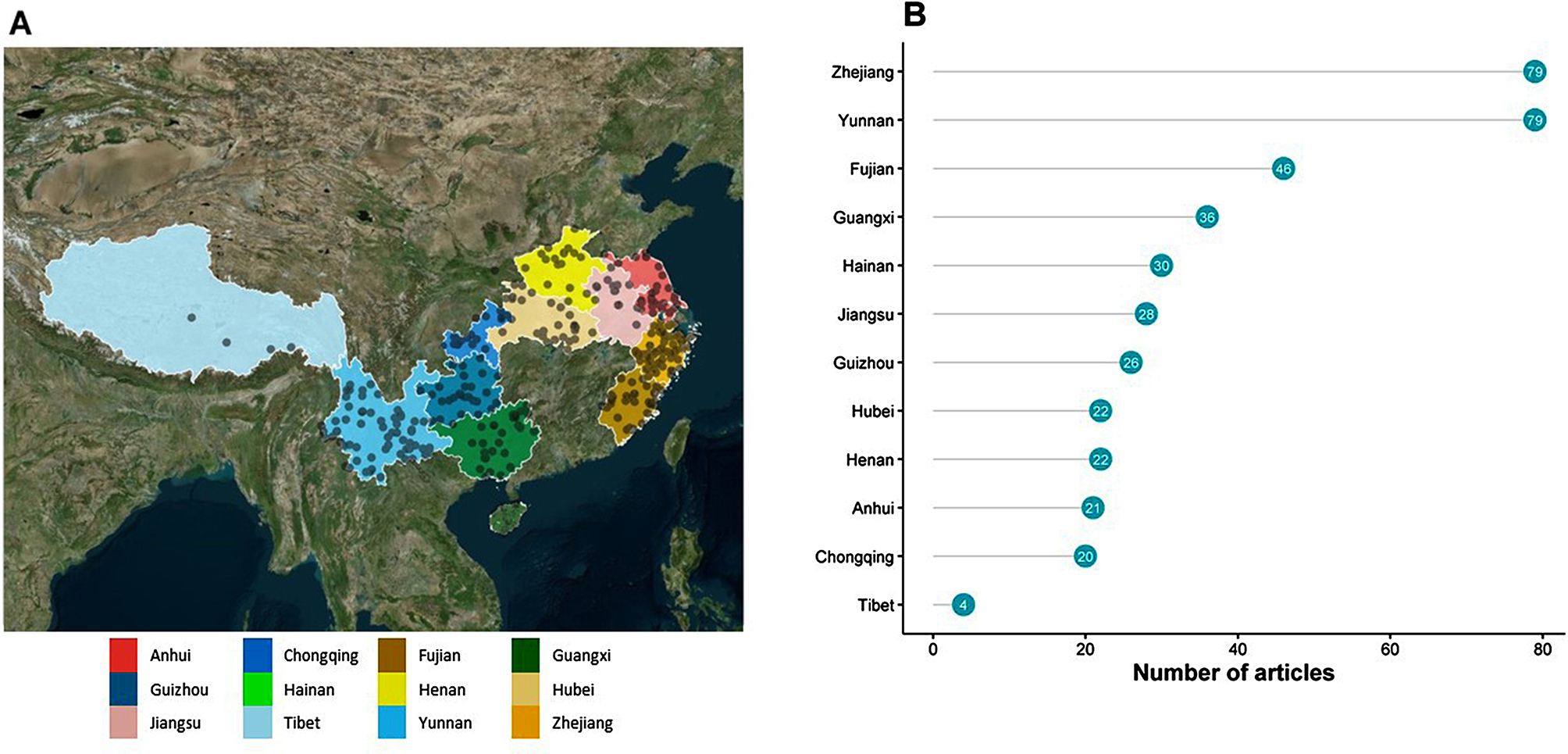
Geographic distribution of published aetiological studies and case reports, in a systematic review of published aetiological studies and case reports from China, 1980–2015. Legend: For panel B, the number of articles will sum to >392 due to some studies being multi-provincial

The majority of included studies were from the south-eastern part of China: 166 (42.3%) records were from Eastern region, 120 (30.6%) from South-Western region, 102 (26.0%) from South-Central region, and 4 (1.0%) were multi-regional studies (Table 2). Yunnan and Zhejiang provinces contributed the highest number of studies (each with *n* = 79 articles), followed in descending order by Fujian (*n* = 46), Guangxi (*n* = 36), and by Hainan, Jiangsu, Guizhou, Anhui, Chongqing, Henan and Hubei (each with ~ 20–40 articles). Tibet contributed a small number (four) of studies (Fig. 2). Yunnan, Hainan, Jiangsu, Hubei, Guizhou, Guangxi and Fujian contributed some studies from the 1980s and 1990s. Overall, there were 19 (4.8%) records published during 1980–1990, 41 (10.5%) during 1991–2000, 211 (53.8%) during 2001–2010 and 121 (30.9%) during 2011–2015 (Supplemental file S2).

**Table 2:** Characteristics of the studies included

	Bacteria (*n* = 154 studies) 1984–2015	Viruses (*n* = 219 studies) 1980–2015	Parasites (*n* = 4 studies) 2007–2012	Fungi (*n* = 1 study) 2009	Bacteria + Viruses (*n* = 13 studies) 2009–2015	Bacteria + Fungi (*n* = 1 study) 2008	Overall (*n* = 392 studies) 1980–2015
**Region**							
Eastern	61 (39.6%)	100 (45.7%)	1 (25.0%)	0 (0.0%)	3 (23.1%)	1 (100.0%)	166 (42.3%)
South-central	47 (30.5%)	46 (21.0%)	0 (0.0%)	1 (100.0%)	8 (61.5%)	0 (0.0%)	102 (26.0%)
South-western	45 (29.2%)	70 (32.0%)	3 (75.0%)	0 (0.0%)	2 (15.4%)	0 (0.0%)	120 (30.6%)
Multi-regional	1 (0.6%)	3 (1.4%)	0 (0.0%)	0 (0.0%)	0 (0.0%)	0 (0.0%)	4 (1.0%)
**Study design**							
Case series	67 (43.5%)	65 (29.7%)	3 (75.0%)	1 (100.0%)	10 (76.9%)	1 (100.0%)	147 (37.5%)
Fever series	23 (14.9%)	10 (4.6%)	0 (0.0%)	0 (0.0%)	0 (0.0%)	0 (0.0%)	33 (8.4%)
Seroprevalence	47 (30.5%)	108 (49.3%)	1 (25.0%)	0 (0.0%)	2 (15.4%)	0 (0.0%)	158 (40.3%)
Multiple study types	17 (11.0%)	36 (16.4%)	0 (0.0%)	0 (0.0%)	1 (7.7%)	0 (0.0%)	54 (13.8%)
**Age distribution**							
Neonates	1 (0.6%)	1 (0.5%)	0 (0.0%)	0 (0.0%)	0 (0.0%)	0 (0.0%)	2 (0.5%)
Children	3 (1.9%)	6 (2.7%)	0 (0.0%)	0 (0.0%)	8 (61.5%)	0 (0.0%)	17 (4.3%)
Adults	43 (27.9%)	27 (12.3%)	3 (75.0%)	1 (100.0%)	1 (7.7%)	0 (0.0%)	75 (19.1%)
All ages	55 (35.7%)	76 (34.7%)	1 (25.0%)	0 (0.0%)	3 (23.1%)	1 (100.0%)	136 (34.7%)
Unspecified	52 (33.8%)	109 (49.8%)	0 (0.0%)	0 (0.0%)	1 (7.7%)	0 (0.0%)	162 (41.3%)
**Sample used**							
Blood	119 (77.3%)	207 (94.5%)	4 (100.0%)	0 (0.0%)	12 (92.3%)	1 (100.0%)	343 (87.5%)
CSF	1 (0.6%)	4 (1.8%)	0 (0.0%)	0 (0.0%)	0 (0.0%)	0 (0.0%)	5 (1.3%)
Blood and/or CSF	30 (19.5%)	8 (3.7%)	0 (0.0%)	1 (100.0%)	1 (7.7%)	0 (0.0%)	40 (10.2%)
Blood or bone-marrow	4 (2.6%)	0 (0.0%)	0 (0.0%)	0 (0.0%)	0 (0.0%)	0 (0.0%)	4 (1.0%)
**Diagnostic Method**							
Culture	42 (27.3%)	1 (0.5%)	0 (0.0%)	1 (100.0%)	0 (0.0%)	1 (100.0%)	45 (11.5%)
Serological	96 (62.3%)	190 (86.8%)	3 (75.0%)	0 (0.0%)	12 (92.3%)	0 (0.0%)	301 (76.8%)
PCR	8 (5.2%)	10 (4.6%)	1 (25.0%)	0 (0.0%)	0 (0.0%)	0 (0.0%)	19 (4.8%)
Culture and/or serological	7 (4.5%)	2 (0.9%)	0 (0.0%)	0 (0.0%)	0 (0.0%)	0 (0.0%)	9 (2.3%)
PCR and/or serological	1 (0.6%)	11 (5.0%)	0 (0.0%)	0 (0.0%)	1 (7.7%)	0 (0.0%)	13 (3.3%)
Unknown	0 (0.0%)	5 (2.3%)	0 (0.0%)	0 (0.0%)	0 (0.0%)	0 (0.0%)	5 (1.3%)

CSF = Cerebrospinal fluid; PCR = polymerase chain reaction; the number in parentheses indicates the column percentages

### Study type and study population

Of the 392 publications included, seroprevalence studies (*n* = 158, 40.3%) and case series (*n* = 147, 37.5%) contributed the majority, with the rest being either fever series (*n* = 33, 8.4%) or study of multiple types (*n* = 54, 13.8%) (Table 2). One third (*n* = 136, 34.7%) included patients of all ages, while around one fifth (19.1%, *n* = 75) included only adults, and very few (*n* = 19, 4.3%) focused solely on children (*n* = 17 among children only and *n* = 2 among neonates only). A sizable proportion (*n* = 162, 41.3%) did not explicitly report the age range of participants (Table 2). Different participant populations were involved, including hospital in-patients (*n* = 183), seroprevalence study conducted in symptomatic individuals (*n* = 56) and in asymptomatic (*n* = 169) individuals, patients presenting with febrile illness (*n* = 45), and in several other settings (*n* = 39) (Percentages add to more than 100 as some studies included participants from multiple groups) (Further details can be found in supplemental file S2).

### Samples collected and diagnostic methods

**Fig. 3:**
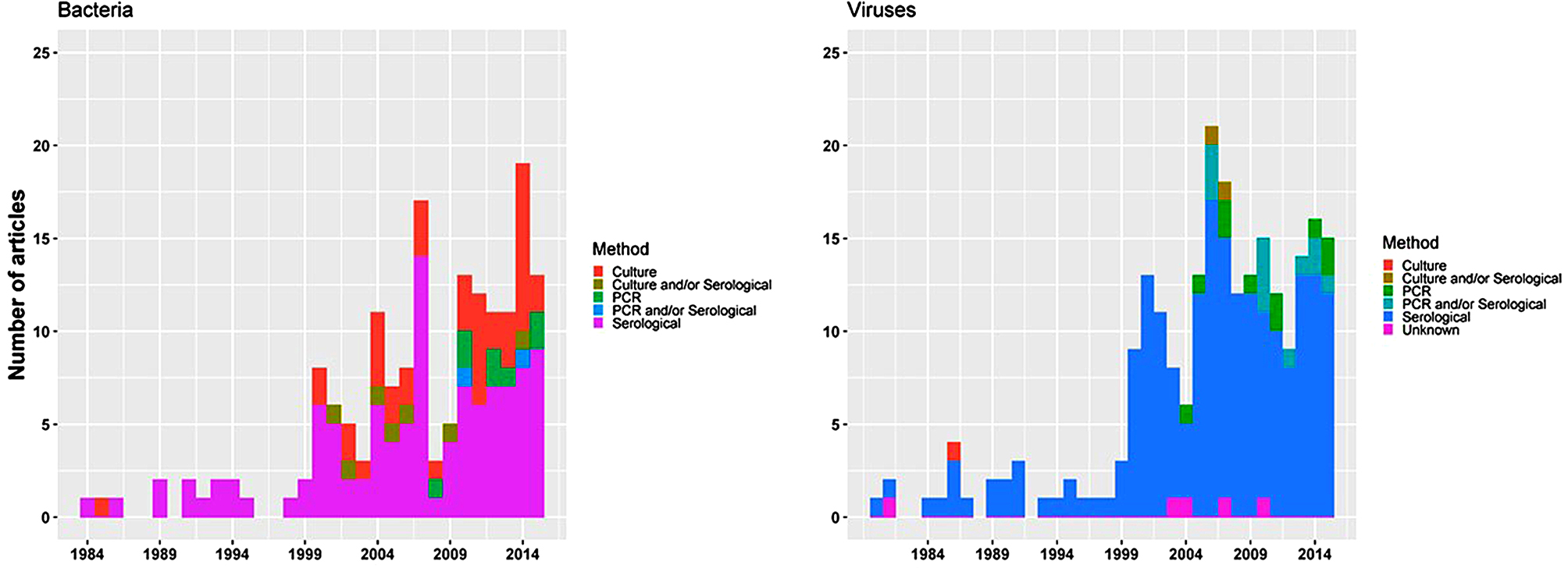
Diagnostics methods used over time-period, in a systematic review of published aetiological studies and case reports from China, 1980–2015

Blood was the most common specimen collected for analysis (*n* = 343/392, 87.5%), followed by cerebrospinal spinal fluid (CSF) (*n* = 5/392, 1.3%) (Table 2). Multiple sample sources, such as blood and/or CSF was used in 40 (10.2%, 40/392) studies and blood/bone-marrow samples in 4 (1.0%, 4/392) studies. Serology was the predominant laboratory approach used for identifying bacterial (*n* = 96/154, 62.3%), viral (*n* = 190/219, 86.8%), and parasitic infections (*n* = 3/4, 75.0%). Culture was the next commonest method used for bacteria (*n* = 42/154, 27.3%), and the sole method used for identifying fungal infections (Table 2). All the identified articles reporting results of polymerase chain reaction (PCR) (*n* = 32/392, 8.2%) were published from 2004 onward. The temporal trends in the use of different laboratory techniques for different pathogens are presented in Fig. 3.

### Aetiological findings

The majority of articles (*n* = 378, 96.4%) reported only a single pathogen group, including viral infections in 219 (55.9%) articles, bacterial infections in 154 (39.3%), parasitic infections in four (1.0%), and fungal infections in one (0.3%). More than one pathogen group was reported in 14 (3.6%) articles, including 13 (3.3%) reporting bacteria and viruses, and 1 (0.3%) reporting bacteria and fungi (Table 2).

### Bacterial infections

**Fig. 4:**
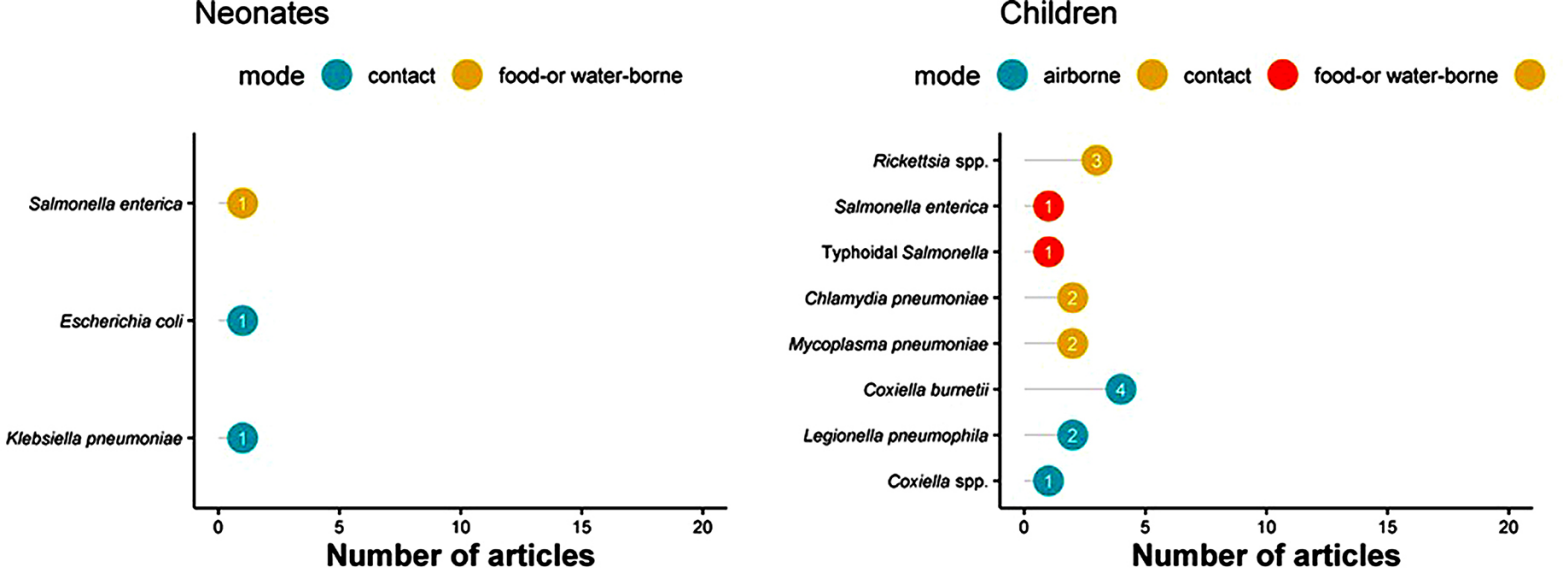
Most commonly reported bacterial infections by predominant mode of transmission in neonates and children, in a systematic review of published aetiological studies and case reports from China, 1980–2015. The numbers inside each dot represent the number of articles. Legend: There were two unique articles reporting results from neonates and 11 from children

**Fig. 5:**
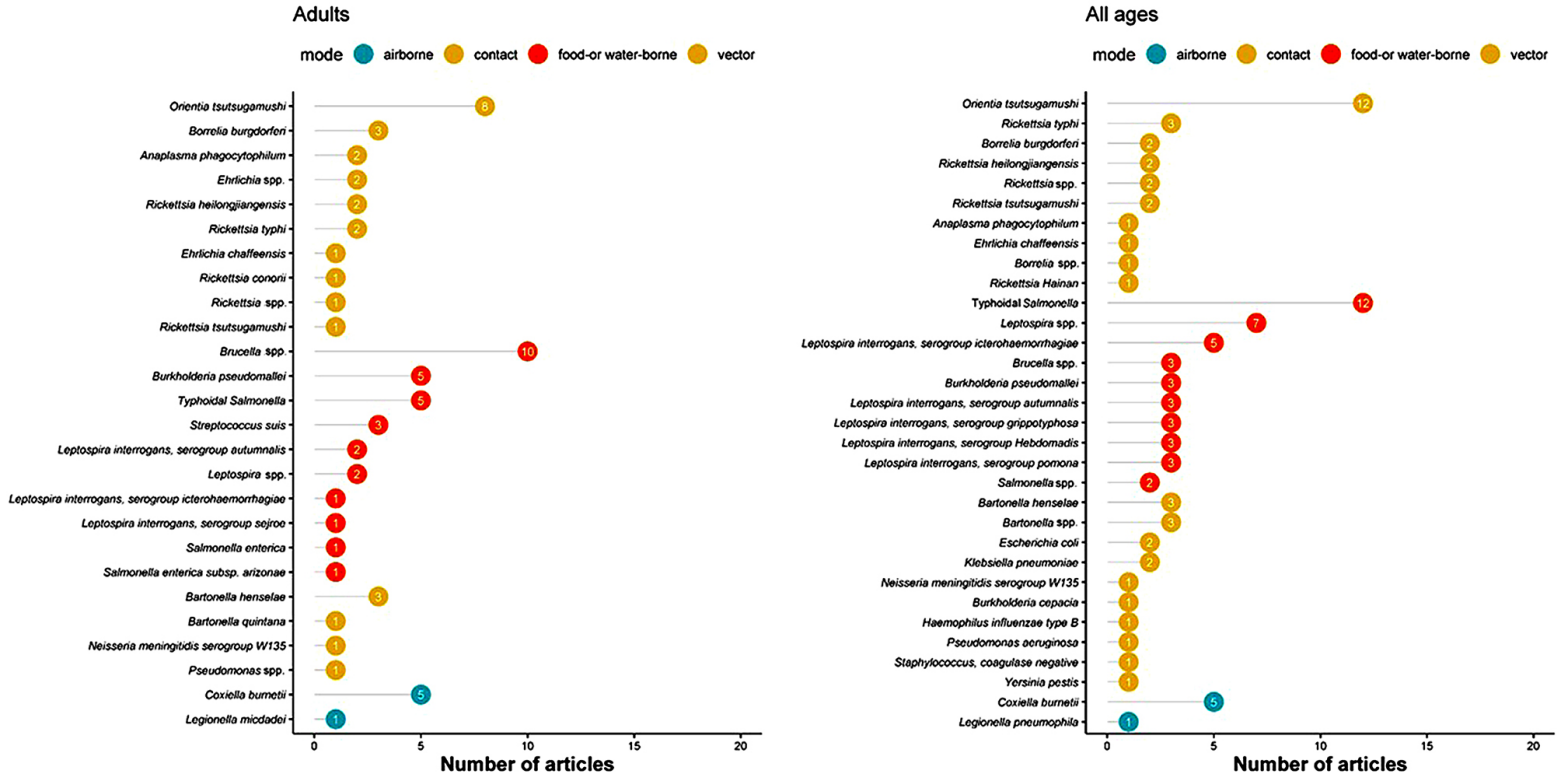
Most commonly reported bacterial infections by predominant mode of transmission in adults and patients of all ages, in a systematic review of published aetiological studies and case reports from China, 1980–2015. The numbers inside each dot represent the number of articles. Legend: There were 46 unique articles reporting results from adults and 59 from studies that enrolled patients of all ages

The top five most commonly reported bacterial pathogens included *Salmonella enterica* serovar Typhi and Paratyphi (*n* = 30), *Orientia/ Rickettsia tsutsugamushi* (*n* = 31), *Coxiella burnetii* (*n* = 17), *Leptospira* spp. (*n* = 15) and *Brucella* spp. (*n* = 15) (Figs. 4 and 5 and Supplemental file S3). Among neonates, *Salmonella enterica, Escherichia coli*, and *Klebsiella pneumoniae* were each reported by one article (Fig. 4; left panel). Among children, *Coxiella burnetii* (*n* = 4), *Rickettsia* spp. (*n* = 3), *Legionella pneumophila* (*n* = 2), *Chlamydia pneumoniae* (*n* = 2), and *Mycoplasma pneumoniae* (*n* = 2) were reported (Fig. 4, right panel). Among older children and adults, the main bacterial infections reported were *Brucella* spp. (*n* = 10) and *Orientia/ Rickettsia tsutsugamushi* (*n* = 9), followed by Typhoidal *Salmonella* (*n* = 5), *Coxiella burnetii* (*n* = 5), and *Burkholderia pseudomallei* (*n* = 5) (Fig. 5 panel A). Overall, there were no substantial differences in the distribution of the bacteria by study design type (case series, fever series or serological studies) (See supplemental 2).

### Identified species of major zoonotic bacterial infections

For vector-borne bacterial infections, rickettsial pathogens were the most commonly reported infection (*n* = 53), including *Orientia/ Rickettsia tsutsugamushi* (*n* = 31), *Rickettsia* spp. (*n* = 9), *Rickettsia typhi* (*n* = 7), *Rickettsia heilongjiangensis* (*n* = 6), *Rickettsia sibirica* (*n* = 6), and *Rickettsia conorii* (*n* = 4), *R. akari* (*n* = 3), *Rickettsia typhi* (*n* = 2), and *Rickettsia hainan* (*n* = 2) (Supplemental files 2 and 3).

### Food- and/or water-borne bacterial infections

For food-and water-borne bacterial infections, Typhoidal Salmonella (*n* = 30 reports) was the leading reported cause, followed by *Leptospira* spp. (*n* = 15) and *Brucella* spp. (*n* = 15). Other reported non-Typhoidal Salmonella included *Salmonella enterica* (*n* = 5), including one paper reporting subsp. Arizonae, and *Salmonella enteritidis* (*n* = 1). The *Leptospira interrogans* serogroups reported included icterohaemorrhagiae (*n* = 8), canicola (*n* = 4), bataviae (*n* = 2), javanica (*n* = 1), and tarassovi (*n* = 1) (Figs. 4 and 5 and Supplemental files 2 and 3).

### Bacterial infections that spread through contact

In the category of contact-related bacterial infections, *Bartonella henselae* (*n* = 8), *Bartonella quintana* (*n* = 5), *Bartonella* spp. (*n* = 4), *Escherichia coli* (*n* = 4), and *Klebsiella pneumoniae* (*n* = 4) were the most commonly reported (Figs. 4 and 5 and Supplemental files 2 and 3).

### Airborne bacterial infections

Airborne bacterial pathogens were reported in 21 articles, including *Coxiella burnetii* in 17 articles, unspecified *Coxiella* spp. in one article, and *Legionella pneumophila* in three, and *Legionella micdadei* in one (Figs. 4 and 5 and Supplemental files 2 and 3).

### Viral infections

**Fig. 6:**
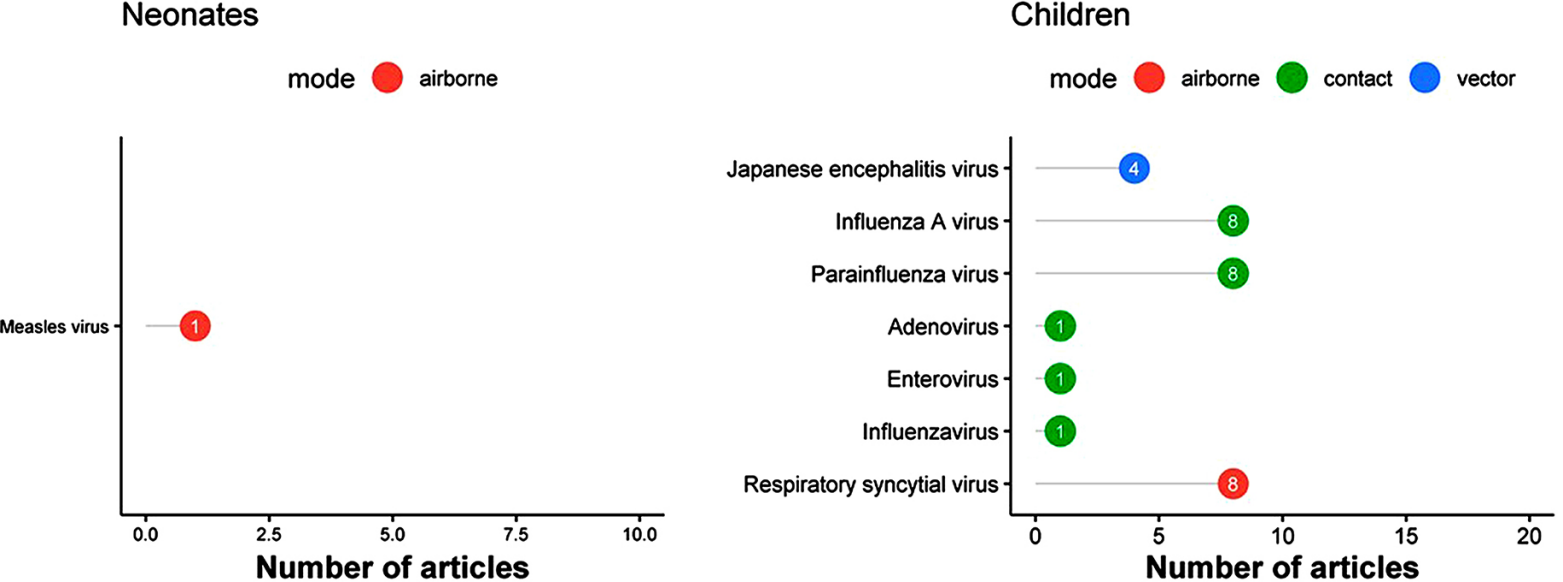
Most commonly reported viral infections by predominant mode of transmission in neonates and children, in a systematic review of published aetiological studies and case reports from China, 1980–2015. The numbers inside each dot represent the number of articles. Legend: There was one unique article reporting results from neonates and 14 from children

**Fig. 7:**
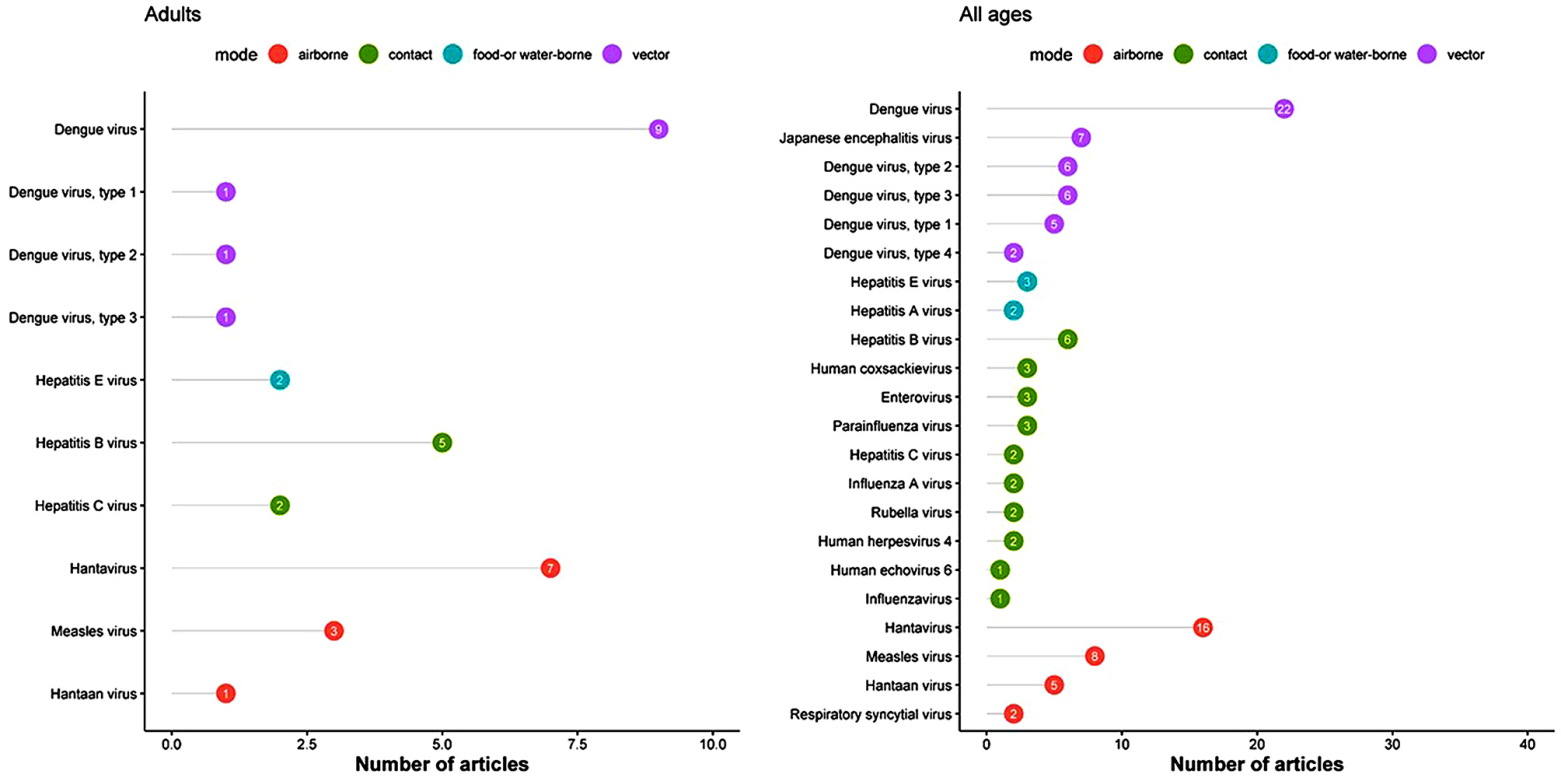
Most commonly reported viral infections by predominant mode of transmission in adults and patients of all ages, in a systematic review of published aetiological studies and case reports from China, 1980–2015. The numbers inside each dot represent the number of articles. Legend: There were 29 unique articles reporting results from adults and 79 from studies that enrolled patients of all ages

Dengue virus (DENV) (*n* = 76 including studies with unclear serotypes), Hantavirus/Hantaan virus (*n* = 89), Japanese encephalitis virus (*n* = 21), and measles virus (*n* = 15) represented the top five most commonly reported viral infections (Figs. 6 and 7 and Supplemental file S3). Among 17 studies reporting infections in children, influenza virus, parainfluenza virus, and respiratory syncytial virus were most commonly reported for children (*n* = 8 for each) (Fig. 6; panel B). In 75 studies that described adult patients, dengue virus (*n* = 9; serovar status unknown), Hantavirus/Hantaan virus (*n* = 8), hepatitis B virus (*n* = 5) and measles virus (*n* = 3) were most commonly reported in adults ( Fig. 7). For neonates, only one paper reporting measles was found (Fig. 6). Overall, there were no substantial differences in the distribution of the viruses by study design type (case series, fever series or serological studies) (See supplemental 2).

### Dengue and other arboviruses

Dengue (DENV) was predominantly reported in studies among adult participants. Overall 76 studies reported DENV, of which 26 studies clearly reported the specific serotypes. Of these 26 studies, DENV2 was reported in 15, DENV3 in 14, DENV1 in 11 and DENV 4 in 4 (the total will add to > 26 as some studies reported multiple serovars) (Supplemental file S2). Other commonly reported non-DENV arboviruses included Japanese encephalitis virus (*n* = 21) and chikungunya virus (*n* = 7), with fewer reports of Murray Valley encephalitis virus (*n* = 2), West Nile virus (*n* = 2), Crimean Congo haemorrhagic fever virus (*n* = 1), Kayasanur Forest disease virus (*n* = 1), Mayaro virus (*n* = 1), Sindbis virus (*n* = 1) and tick-borne encephalitis virus (*n* = 1) (Supplemental files 2 and 3).

**Fig. 8:**
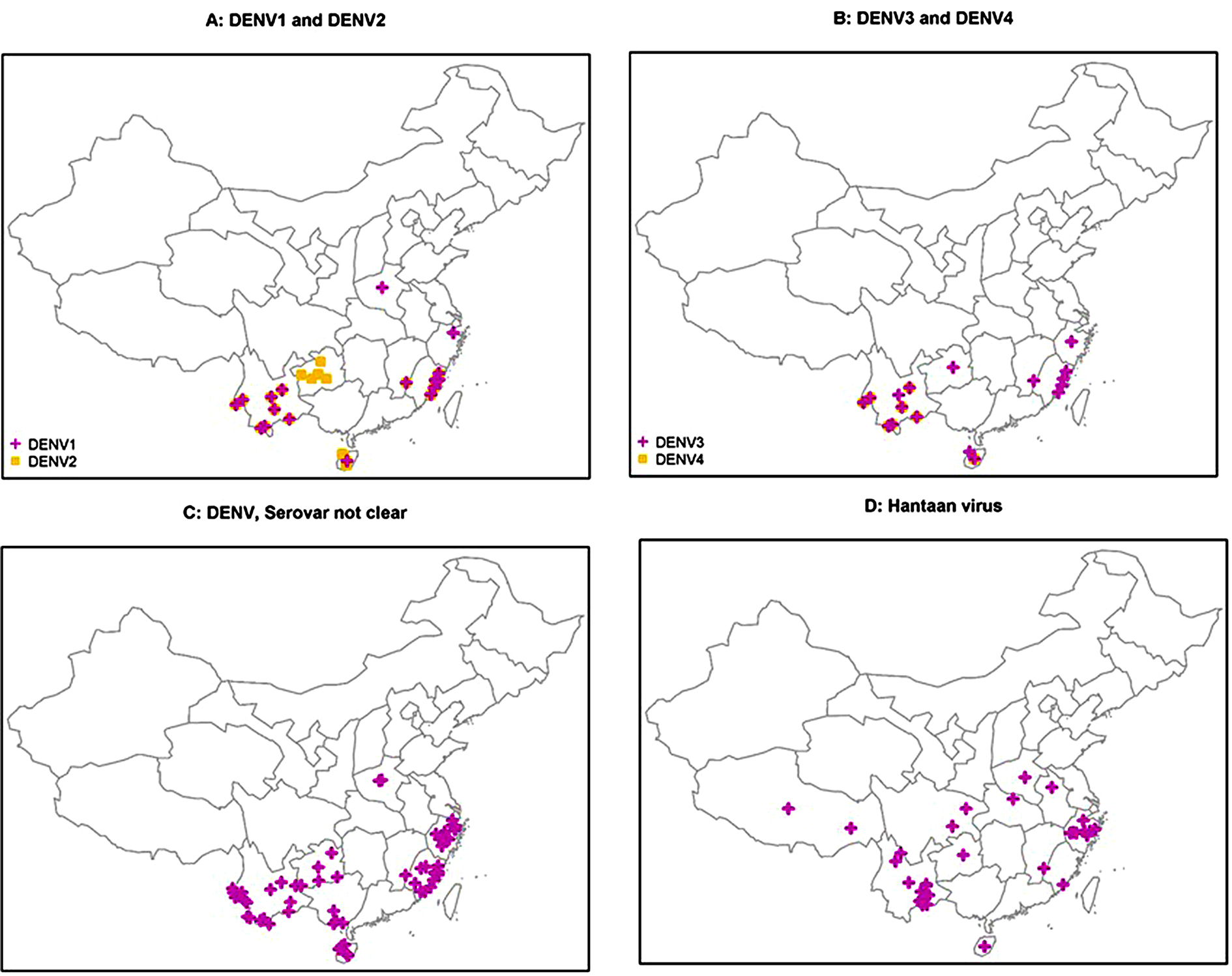
Reports of Dengue virus and Hantaan virus, in a systematic review of published aetiological studies and case reports from China, 1980–2015

A distinct geographical pattern existed for arboviruses. Dengue virus (DENV) infections were reported only from seven provinces in China during the period reviewed, including Zhejiang (*n* = 16), Fujian (*n* = 15), Henan (*n* = 2), Guangxi (*n* = 4), Guizhou (*n* = 7), Yunnan (*n* = 26), and Hainan (*n* = 11), which are mostly along coastal and inland national border areas. For different DENV serotypes, DENV1 were reported from Fujian (*n* = 4), Henan (*n* = 1), Yunnan (*n* = 5), and Zhejiang (*n* = 1); DENV2 was reported from Guizhou (*n* = 1), Hainan (*n* = 6), Fujian (*n* = 4), and Yunnan (*n* = 5); DENV3 from Fujian (*n* = 3), Guizhou (*n* = 1), Zhejiang (*n* = 3), Yunnan (*n* = 4), and Hainan (*n* = 4); and DENV4 from Yunnan (*n* = 3) and Hainan (*n* = 1) (Fig. 8). For other non-DENV arboviruses, Hantavirus/Hantaan virus infection was reported most commonly from Zhejiang (*n* = 30) and Yunnan (*n* = 19). Crimean Congo haemorrhagic fever was only reported from Henan (*n* = 1); while Kyasanur Forest disease virus was only reported form Guizhou (*n* = 1). For other infections, there were either no specific geographical pattern or insufficient numbers of reports for assessing geo-temporal trends (Supplemental files 2 and 3).

### Food- and/or water-borne viral infections

Hepatitis E virus was reported in six articles and Hepatitis A in three, all in adults (Supplemental file S3).

### Airborne viral infections

Hantavirus/Hantaan virus was reported in 89 articles, geographically distributed widely across the southern parts of China. Measles virus was reported in 15 articles. Respiratory syncytial virus (RSV) was reported in 11 articles. No other airborne viruses were reported (Supplemental file S2 and 3).

### Viral infections spreading through contact

Sixteen different contact transmissible viruses were reported in a total of 47 unique articles. The most commonly reported viral infections included influenza A (*n* = 12 reports), parainfluenza (*n* = 11), hepatitis B (*n* = 11) and hepatitis C (*n* = 3) (Supplemental files S2 and S2).

### Viral infections in neonates and children

Only one paper specifically examined viruses in neonates, reporting on measles virus (Fig. 6). In children, the following viruses were reported: influenza A virus (*n* = 8), parainfluenza virus (*n* = 8), respiratory syncytial virus (*n* = 8), Japanese encephalitis virus (*n* = 4) and influenza virus (*n* = 1) (Fig. 6).

### Parasitic and fungal infections

Only 4 (1.0%) papers specifically reported on non-malarial parasitic infections and 2 (0.5%) reported fungal infections over the study period. These included four papers on parasitic infections, reporting *Leishmania* spp. (*n* = 2) and *Babesia* spp. (*n* = 1) for vector-borne, and *Echinococcus* spp. (*n* = 1), *Toxoplasma gondii* (*n* = 1), C*lonorchis sinensis* (*n* = 1), and tapeworm larva (*n* = 1) for food-or-water-borne parasitic infections. For fungi, only one paper reported *Talaromyces (Penicillium) marneffei* infection (*n* = 1) and another article reported an unknown fungus (*n* = 1) (Supplemental files 2 and 3).

### Temporal trends in infectious causes of fever

Temporal trend in most commonly reported pathogens is presented in Table 3. *Salmonella* spp. were reported for most of the study period. *Borrelia burgdorferi* (Lyme disease) was reported in 4 articles during 2001–2010 and in an article during 2011–2015. *Burkholderia pseudomallei* (Melioidosis) was reported in 6 articles during 2001–2010 and 3 articles published during 2011–2015 while *Streptococcus suis* was reported in two articles (2001–2010). Hantavirus and dengue virus were reported throughout the study periods whereas the limited number of studies describing parasites and fungi were published during 2001–2010 period (Table 3 and supplemental file S2).

**Table 3:** Commonly reported pathogens by time-period

	1980 to ≤ 1990 (*n* = 19)	1991 to ≤ 2000 (*n* = 41)	2001 to ≤ 2010 (*n* = 211)	2011 to ≤ 2015 (*n* = 121)
**Bacteria**	*Coxiella burnetii* (*n* = 4)	*Rickettsia sibirica* (*n* = 5)	Typhoidal *Salmonella* (*n* = 17)	*Brucella* spp. (*n* = 12)
	Typhoidal *Salmonella* (*n* = 2)	*Borrelia* spp. (*n* = 4)	*Orientia tsutsugamushi* (*n* = 15)	Typhoidal *Salmonella* (*n* = 11)
	-	*Rickettsia conorii* (*n* = 3)	*Leptospira* spp. (*n* = 12)	*Coxiella burnetii* (*n* = 9)
	-	*Rickettsia akari* (*n* = 3)	*Leptospira interrogans*, *serogroup icterohaemorrhagiae* (*n* = 6)	*Orientia tsutsugamushi* (*n* = 8)
	-	*Orientia tsutsugamushi* (*n* = 3)	*Burkholderia pseudomallei* (*n* = 6)	*Rickettsia typhi* (*n* = 5)
		*Salmonella enterica (n = 2)*	*Leptospira interrogans, serogroup grippotyphosa (n = 5)*	*Rickettsia* spp. (*n* = 5)
**Viruses**	Hantavirus (*n* = 6)	Hantavirus (*n* = 12)	Hantavirus (*n* = 44)	Dengue virus (*n* = 24)
	Dengue virus, type 2 (*n* = 4)	Japanese encephalitis virus (*n* = 4)	Dengue virus (*n* = 31)	Hantavirus (*n* = 12)
	Dengue virus, type 3 (*n* = 3)	Dengue virus (*n* = 4)	Measles virus (*n* = 10)	Respiratory syncytial virus (*n* = 10)
	Japanese encephalitis virus (*n* = 2)	Chikungunya virus (*n* = 3)	Hepatitis B virus (*n* = 8)	Parainfluenza virus (*n* = 10)
	Chikungunya virus (*n* = 2)	Hepatitis B virus (*n* = 2)	Hantaan virus (*n* = 8)	Japanese encephalitis virus (*n* = 9)
**Parasites**	-	-	*Leishmania* spp. (*n* = 2)	*Babesia* spp. (*n* = 1)
	-	-	*Toxoplasma gondii* (*n* = 1)	-
	-	-	Tapeworm larva (*n* = 1)	-
	-	-	*Echinococcus* spp. (*n* = 1)	-
	-	-	*Clonorchis sinensis* (*n* = 1)	-
**Fungi**	-	-	*Penicillium marneffei* (n = 1)	-
	-	-	Fungus (unknown) (*n* = 1)	-

The number in parentheses indicates the number of publications reporting the given microorganism

### Assessment of risk of bias

The large majority of studies included in this review were considered to have either a moderate (*n* = 229, 58.4%) or high (*n* = 149, 38.0%) risk of bias, with only 3.6% (*n* = 14) considered to be of low risk (Supplemental file 2). The geographical pattern of the data availability also suggests an issue of reporting bias in relation to differential awareness, and ascertainment in different provinces, probably in relation to their different socio-economic and academic publishing development.

## Discussion

This systematic review presents a landscape of infectious causes of non-malarial febrile illness reported over the last four decades in malarious provinces of China. During this period, malaria transmission has significantly declined, making the findings of this review particularly relevant and timely for guiding updates to clinical management guidelines, public health interventions, and future research and surveillance on infectious causes of febrile illness [3].

This review identified a broad range of pathogens recognized in normally sterile sites or detected by serology. We also presented the distribution of the pathogens by mode of transmission which can potentially inform future control measures. Overall, most of the reported infections identified in this review are well known and commonly encountered in countries at all income levels. Overall, viral and bacterial infections represented the two most commonly reported pathogen groups, with reports on these groups constituting > 95% of the articles included. Well-known bacterial pathogens, including *Rickettsia, Salmonella, Coxiella, Leptospira*, and *Bartonella*, were commonly reported. Reports of fungal and parasitic infections were rare. This pattern of predominance of bacterial and viral pathogens was compatible with findings based mainly on national notifiable disease and surveillance data [20]. Some infections commonly reported in national notifiable disease and surveillance data, such as tuberculosis and HIV/AIDS, were excluded from this review as they do not typically present with a febrile clinical picture overlapping with that of malaria [20]. The over-representation of studies from the south and eastern part of the country contrasted with the disproportionate burden of infectious diseases borne by children and adolescents in western China [20], may reflect the presence of research, research activities and/or reporting bias.

There was a relative scarcity of reports focused only on children (4.8%), although children may have been included in other articles with participants of all ages, or with age ranges not reported. For children and neonates, common viral infections like influenza virus, parainfluenza virus, respiratory syncytial virus, and measles were reported most frequently. The common reports of seasonal influenza infection in this age group was consistent with observations from surveillance data [20]. For adults, however, potentially severe arboviral infections like dengue virus and hantavirus infections were disproportionately frequently reported, possibly reflecting a reporting and publication bias towards potentially lethal infections of major outbreak potential. The reporting of all dengue serotypes in the country was compatible with findings reported from other studies on dengue epidemiology [21, 22]. The frequent reporting of viral hepatitis is also compatible with its recognition as a major health care burden in China, with an estimated hepatitis B virus prevalence of 5–8% in the general population, with more than 90% in adults older than 20 years [23].

### Limitations

Adhering to a rigorous methodological approach, our review included relevant studies identified from three English-language and three Chinese-language databases, published over a period of 36 years from 1980 to 2015. Public access to the results is allowed by the functionality of interactive visualization and maps (https://www.iddo.org/surveyor/NMFI/#0). A major advantage is the inclusion of publications in Chinese language. However, our review shares several of the limitations discussed in the preceding articles in this series [8–10]. Firstly, studies included exhibited huge heterogeneity in terms of study design, patient population, and laboratory/diagnostic testing procedures. Reporting of important data, including participants’ age, sex, source of the sample/s used to diagnose the infection, and case definitions was also neither consistent nor systematically reported. In consequence, a large proportion (96.4%) of the studies included in this review were considered to be at moderate to high risk of bias. This precluded the possibility for any meaningful amalgamation and meta-analysis of the data. We also did not include grey literature in this review. As the current review is mainly focused on describing detection of infections, some data, including antimicrobial susceptibility/resistance patterns, and specific antibody subtypes (IgM/ IgG) tested in a seroprevalence study, were not collected and reported in this review, even if they were reported in some articles.

Results of temporal and geographical patterns of reported infections must be interpreted with great care, and may not represent the true picture of pathogen distribution. While positive reports should indicate the presence of a pathogen, the lack of reporting of a pathogen from a county or region cannot be taken as definitive evidence of its absence in that setting. Many factors may affect whether a particular pathogen was tested for and reported in the literature. These may include awareness and prior recognition, clinical suspicion, experience and expertise of health care workers, facility availability, competing service needs, sampling and testing practice, prior exposure and treatment, research development, academic interest, and specific research questions addressed, among other considerations. As our data were quantified by the unit of published articles rather than frequency of isolation of pathogens or disease episodes, our results cannot be used for inferring the incidence or prevalence of pathogens or infections. Publication and reporting bias on potentially severe, new, or unusual pathogens may also substantially distort the published literature. Observed temporo-spatial patterns of infections reported in this review, therefore, should not be over-interpreted as reflecting definite patterns of disease incidence or pathogen prevalence.

Our restriction to articles reporting organisms identified by serology and organisms isolated from normally sterile sites, the latter necessary to avoid confusion of colonization from true infection, may have limited the inclusion of some important and/or common pathogens, such as helminths, respiratory infections, and sexually transmitted infections. Routine and systemic laboratory data on clinical specimens being tested in hospitals and private laboratories may provide a more consistent representation of the common causes of non-malaria febrile illness in a region, although they are rarely available in scientific literature.

### Implications for policymakers and other stakeholders

The findings from this review carry important implications for different stakeholders in the health care system. The pattern of pathogens identified in different regions of China could provide a framework for updating clinical guidelines for health workers caring for local residents and regional and international travellers to different areas in China. These findings could also support evidence-based recommendations for vaccines, prophylactic antimicrobial medications, clinical diagnostic services, and future surveillance and research efforts.

The heterogeneous and patchy reporting of many infectious diseases, on the other hand, highlights the need to improve the implementation and coordination of suitable data reporting systems on different geographical levels, and effective and consistent utilization of relevant routine clinical and laboratory data, to inform systemic and continuous disease surveillance [24]. The great variability in diagnostic and reporting practices highlights the importance of efforts on standardizing important key operational steps and protocols, such as specimen collection and diagnostic methods, typing methods and classification systems, antimicrobial susceptibility profiles, and vaccination practices, which would help to better make sense of observed temporal and geographical patterns of disease occurrence and health care utilization burden of NMFI. The recently published Microbiology Investigation Criteria for Reporting Objectively (MICRO) guideline represents one such example [25]. A way forward to enhance understanding would be accessible systems to link surveillance data with research data, academic and lay, endemic and epidemic, to inform policy and implementation [26].

The remarkable underrepresentation of reporting, either positive or negative, from western China reflects potential gaps in diagnostic and research infrastructure and capability in those regions, where more resources could be invested and expertise built in order to inform infection surveillance and reporting in a manner more consistent with other areas of the country [27].

## Conclusion

This review provides a comprehensive summary of reports on non-malarial causes of febrile illness in China published during 1980–2015. Our results demonstrate substantial heterogeneity, inconsistent reporting, and areas with considerable gaps where more resources should be allocated and capacity development initiatives put in place. The majority (> 95%) of the published articles reported on bacteria and viruses as potential infectious aetiologies of fever in China, with comparatively little reporting of fungi and parasites. There were also relatively few reports regarding infectious causes of febrile illnesses in neonates, infants and children. Our findings highlight the need to (i) standardize protocols and guidelines for fever aetiology studies for better comparability of results and interpretation, (ii) improve existing epidemiological surveillance networks to inform global fever policy priorities, and (iii) prioritize the development of pathogen-target diagnostics and implementation of fever-testing algorithms in the country.

### Electronic supplementary material

Below is the link to the electronic supplementary material.

Supplementary Material 1

Supplementary Material 2

Supplementary Material 3

## Data Availability

All data generated and analysed in this review are openly available from the IDDO webpage as a downloadable resource (https://www.iddo.org/febrile-illness).

## Abbreviations

CDC: US Centers for Disease Control and Prevention
CNKI: China National Knowledge Infrastructure
CSF: Cerebrospinal fluid
IDDO: Infectious Diseases Data Observatory
MeSH: Medical Subject Headings
MICRO: Microbiology Investigation Criteria for Reporting Objectively
NMEP: National Malaria Elimination Programme
NMFI: Non-Malarial Febrile Lllness
PCR: Polymerase Chain Reaction
PRISMA: Preferred Reporting Items for Systematic Reviews and Meta-Analyses
RDT: Rapid Diagnostic Test
WHO: World Health Organization
