# Supplementary material for: Non-malarial febrile illness: a systematic review of published aetiological studies and case reports from China, 1980–2015
Source: BMC Infect Dis. 2024 Aug 20;24:843. doi: 10.1186/s12879-024-09542-3 (PMC11334328; doi:10.1186/s12879-024-09542-3)
Supplement: Supplementary file 1 — Supplementary Material 1 [file 12879_2024_9542_MOESM1_ESM.docx]

**Supplemental file 1: Search terms and keywords adopted for**

**Non-malarial febrile illness: a systematic review of published aetiological studies and case reports from China, 1980-2015**

Dennis K. M. Ip^*^, Yvonne Y. Ng, Yat H. Tam, Nigel V. Thomas, Prabin Dahal, Kasia Stepniewska, Paul N. Newton, Philippe J. Guérin, Heidi Hopkins^*^

*[dkmip@hku.hk](mailto:dkmip@hku.hk)

*[Heidi.Hopkins@lshtm.ac.uk](mailto:Heidi.Hopkins@lshtm.ac.uk)

| 1. **China: Ovid MEDLINE(R) In-Process & Other Non-Indexed Citations <May 03, 2016>, Ovid MEDLINE(R) <1946 to April Week 3 2016> (Searched May 04, 2016)** | |
| --- | --- |
| 1 | exp Anti-Bacterial Agents/dt, pd, tu, th, ut [Drug Therapy, Pharmacology, Therapeutic Use, Therapy, Utilization](409435) |
| 2 | ext Bacteremia/ or (bacteremi* or bacteraemi* or septicemi* or septicaemi*).ti,ab.(42887) |
| 3 | exp Sepsis/ or ("blood stream infection*" or "bloodstream infection*" or "blood stream pathogen*" or "bloodstream pathogen*" or (("blood borne" or bloodborne or blood-borne) adj (infection* or pathogen*))).ti,ab.(107115) |
| 4 | exp Fever/ or (febrile or fever* or pyrexia or pyrexic or pyrexial or pyrexiae or hyperthermi* or (raised adj2 temperature) or (high adj 2 temperature) or (elevated adj2 temperature)).ti,ab.(199437) |
| 5 | or/1-3 (517826) |
| 6 | (babesi* or leishmania* or chagas or trypanosom* or "sleeping sickness").ti,ab.(63311) |
| 7 | Babesia microti/(241) |
| 8 | exp Trypanosoma/(22703) |
| 9 | exp Trypanosomiasis/ not Trypanosomiasis, Bovine/(18477) |
| 10 | exp Leishmania/(16762) |
| 11 | exp Leishmaniasis/(18938) |
| 12 | Influenza A Virus, H1N1 Subtype/ or Influenza A virus/ or Influenza a virus.ti,ab.(31980) |
| 13 | exp Arbovirus infections/ or Arboviruses/ or exp Encephalitis viruses, Japanese/ or Encephalitis virus, Japanese/ or Encephalitis virus, Murray Valley/ or "Encephalitis virus, St. Louis"/ or West Nile virus/ or Encephalitis viruses, tick-borne/ or Yellow fever virus/ or (Japanese Encephalitis or Japanese B Encephalitis or Phlebotomus fever or Rift Valley fever or Yellow fever or ((California or "St Louis" or Tick-borne) adj Encephalitis) or Kyasanur Forest disease).ti,ab.(39546) |
| 14 | Chikungunya virus/ or Chikungunya.ti,ab.(2699) |
| 15 | exp Epstein-Barr Virus Infections/ or Epstein-Barr.ti,ab.(42804) |
| 16 | exp Dengue/ or Dengue Virus/ or Dengue.ti,ab.(15118) |
| 17 | Respiratory Syncytial Virus, Human/ or Respiratory Syncytial Virus Infections/ or (Respiratory Syncytial Virus or RSV).ti,ab.(14080) |
| 18 | Measles/ or Measles virus/ or Measles.ti,ab.(22978) |
| 19 | Hantavirus/ or Hantaan Virus/ or Puumala Virus/ or Seoul Virus/ or Sin Nombre Virus/ or Hantavirus Infections/ or Hantavirus Pulmonary Syndrome/ or Hemorrhagic Fever with Renal Syndrome/ or (Hantavirus or Hantaan or Puumula or Seoul virus or Sin Nombre virus or Hemorrhagic fever or Haemorrhagic fever).ti,ab.(10371) |
| 20 | Nipah Virus/ or Nipah Virus.ti,ab.(606) |
| 21 | Coxsackievirus Infections/ or (Coxsackie or Coxsackievirus*).ti,ab.(8791) |
| 22 | Cytomegalovirus Infections/ or Cytomegalovirus/ or Cytomegalovirus*.ti,ab.(43305) |
| 23 | Hepatitis/ or Hepatitis, Viral, human/ or Hepatitis a/ or Hepatitis b/ or Hepatitis b, chronic/ or Hepatitis c/ or Hepatitis c, chronic/ or Hepatitis e/ or Hepatitis b virus/ or Hepacivirus/ or Hepatitis e virus/ or Hepatitis a virus/ or Hepatitis a virus, human/ or (Hepatitis adj (a or b or c or e)).ti,ab.(179013) |
| 24 | (Coxiella burnetii or Salmonella typhi or Salmonella paratyphi or Burkholderia pseudomallei or Pseudomonas pseudomallei or Brucella or Escherichia coli or E-coli or E coli or Citrobacter freundii or Listeria monocytogenes or (Leptospira adj (kmetyi or interogans or weilii or parva)) or Rickettsia or Orientia tsutsugamushi or mycobacterium tuberculosis or h1n1 or Human Herpesvirus 4 or Human Herpes virus 4 or Ehrlichia or Anaplasma or Bartonella or Borrelia or Neorickettsia or Sennetsu or Blastomyces or Cryptococcus or Coccidioides or Histoplasma or Penicillium marneffii or Talaromyces or Yersinia pestis or Francisella tularensis or Klebsiella pneumoniae or Mycoplasma).ti,ab.(401698) |
| 25 | (Q fever or salmonellosis or salmonella infection* or melioidosis or brucellosis or listeria infection* or listeriosis or leptospira* infection* or leptospirosis or rickettsia* infection* or rickettsios* or enteric fever or typhoid fever or paratyphoid fever or plague or anaplasmosis or ehrlichiosis or cat scratch fever or cat scratch disease or trench fever or Carrion* disease or borreliosis or lyme or bartonellosis or relapsing fever or typhus or blastomycosis or cryptococcal or coccidioidomycosis or valley fever or histoplasmosis or penicilliosis or tularaemia or tularemia).ti,ab.(81191) |
| 26 | Blood-Borne Pathogens/ (2721) |
| 27 | ((gram-positive adj3 (bacteria* or infection*)) or (gram-negative adj3 (bacteria* or infection*))).ti,ab. (43378) |
| 28 | Cerebrospinal Fluid/ or (cerebrospinal fluid or csf).ti,ab. (128268) |
| 29 | exp Bacterial Infections/ not exp Bacteremia/ (768745) |
| 30 | exp Bacteria/ (1168342) |
| 31 | or/6-30(2241002) |
| 32 | exp Animals/ (20097744) |
| 33 | Humans/ (15868164) |
| 34 | 32 not (32 and 33)(4229580) |
| 35 | exp China/ or China.ti,ab.(158566) |
| 36 | (Anhui or Fujian or Guizhou or Hainan or Henan or Hubei or Jiangsu or Yunnan or Zhejiang or Guangxi or Tibet or Xizang or Chongqing).ti,ab.(16518) |
| 37 | or/35-36(161723) |
| 38 | 5 and 31 and 37(2191) |
| 39 | 4 and 37(2374) |
| 40 | 38 or 39(4456) |
| 41 | 40 not 34(3992) |
| 42 | limit 41 to (chinese or english or french or portuguese or spanish)(3942) |
| 43 | limit 42 to yr="1980-Current"(3885) |

| **2. China: Global Health <1910 to 2016 Week 16> (Searched May 4, 2016)** | |
| --- | --- |
| 1 | exp antibacterial agents/(86422) |
| 2 | (bacteremi* or bacteraemi* or septicemi* or septicaemi*).ti,ab.(17972) |
| 3 | bacteraemia/(10483) |
| 4 | ("blood stream infection*" or "bloodstream infection*" or "blood stream pathogen*" or "bloodstream pathogen*" or sepsis or (("blood borne" or bloodborne or blood-borne) adj (infection* or pathogen*))).ti,ab.(21333) |
| 5 | sepsis/(11897) |
| 6 | or/1-5(118832) |
| 7 | (febrile or fever* or pyrexia or pyrexic or pyrexial or pyrexiae or hyperthermi* or (raised adj2 temperature) or (high adj 2 temperature) or (elevated adj2 temperature)).ti,ab.(100254) |
| 8 | fever/(23132) |
| 9 | or/7-8(100662) |
| 10 | (babesi* or leishmania* or chagas or trypanosom* or "sleeping sickness").ti,ab.(70850) |
| 11 | babesia microti/(693) |
| 12 | exp trypanosoma/(38996) |
| 13 | exp Trypanosomiasis/ not Trypanosomiasis, Bovine/(18521) |
| 14 | exp Leishmania/(33233) |
| 15 | exp Leishmaniasis/(21442) |
| 16 | Influenza A Virus, H1N1 Subtype/ or Influenza A virus/ or Influenza a virus.ti,ab.(12653) |
| 17 | exp Arbovirus infections/ or Arboviruses/ or exp Encephalitis viruses, Japanese/ or Encephalitis virus, Japanese/ or Encephalitis virus, Murray Valley/ or "Encephalitis virus, St. Louis"/ or West Nile virus/ or Encephalitis viruses, tick-borne/ or Yellow fever virus/ or (Japanese Encephalitis or Japanese B Encephalitis or Phlebotomus fever or Rift Valley fever or Yellow fever or ((California or "St Louis" or Tick-borne) adj Encephalitis) or Kyasanur Forest disease).ti,ab.(28001) |
| 18 | Arboviruses/ or exp Encephalitis viruses, Japanese/ or Encephalitis virus, Japanese/ or Encephalitis virus, Murray Valley/ or "Encephalitis virus, St. Louis"/ or West Nile virus/ or Encephalitis viruses, tick-borne/ or Yellow fever virus/ or (Japanese Encephalitis or Japanese B Encephalitis or Phlebotomus fever or Rift Valley fever or Yellow fever or ((California or "St Louis" or Tick-borne) adj Encephalitis) or Kyasanur Forest disease).ti,ab.(28001) |
| 19 | japanese encephalitis/ or st louis encephalitis/ or tickborne encephalitis/(4869) |
| 20 | west nile virus/(5023) |
| 21 | Murray Valley encephalitis virus/(496) |
| 22 | st louis encephalitis virus/(1400) |
| 23 | Japanese encephalitis virus/(3812) |
| 24 | St Louis encephalitis/(185) |
| 25 | sandfly fever/ or "sandfly fever (naples)"/ or "sandfly fever (sicilian)"/(713) |
| 26 | exp arboviruses/(8168) |
| 27 | Rift Valley fever/ or Rift Valley fever virus.od.(1593) |
| 28 | yellow fever/ or yellow fever virus/(6848) |
| 29 | california encephalitis virus/(1009) |
| 30 | tickborne encephalitis/ or tick-borne encephalitis virus/(2343) |
| 31 | (Japanese Encephalitis or Japanese B Encephalitis or Phlebotomus fever or Rift Valley fever or Yellow fever or ((California or "St Louis" or Tick-borne) adj Encephalitis) or Kyasanur Forest disease).ti,ab.(18850) |
| 32 | Chikungunya virus/ or Chikungunya.ti,ab.(2644) |
| 33 | human herpesvirus 4/ or Epstein-Barr.ti,ab.(6296) |
| 34 | exp Dengue/ or Dengue Virus/ or Dengue.ti,ab.(16167) |
| 35 | Respiratory Syncytial Virus, Human/ or Respiratory Syncytial Virus Infections/ or (Respiratory Syncytial Virus or RSV).ti,ab.(5346) |
| 36 | Measles/ or Measles virus/ or Measles.ti,ab.(13021) |
| 37 | Hantavirus/ or Hantaan Virus/ or Puumala Virus/ or Seoul Virus/ or Sin Nombre Virus/ or Hantavirus Infections/ or Hantavirus Pulmonary Syndrome/ or Hemorrhagic Fever with Renal Syndrome/ or (Hantavirus or Hantaan or Puumula or Seoul virus or Sin Nombre virus or Hemorrhagic fever or Haemorrhagic fever).ti,ab.(8374) |
| 38 | Nipah Virus/ or Nipah Virus.ti,ab.(531) |
| 39 | coxsackieviruses/ or (Coxsackie or Coxsackievirus*).ti,ab.(3916) |
| 40 | cytomegalovirus/ or Cytomegalovirus*.ti,ab.(11312) |
| 41 | hepatitis a/ or hepatitis a virus/ or hepatitis b/ or hepatitis b virus/ or hepatitis c/ or hepatitis c virus/ or hepatitis e/ or hepatitis e virus/ or hepatitis e-like viruses/ or (Hepatitis adj (a or b or c or e)).ti,ab.(59032) |
| 42 | (Coxiella burnetii or Salmonella typhi or Salmonella paratyphi or Burkholderia pseudomallei or Pseudomonas pseudomallei or Brucella or Escherichia coli or E-coli or E coli or Citrobacter freundii or Listeria monocytogenes or (Leptospira adj (kmetyi or interogans or weilii or parva)) or Rickettsia or Orientia tsutsugamushi or mycobacterium tuberculosis or h1n1 or Human Herpesvirus 4 or Human Herpes virus 4 or Ehrlichia or Anaplasma or Bartonella or Borrelia or Neorickettsia or Sennetsu or Blastomyces or Cryptococcus or Coccidioides or Histoplasma or Penicillium marneffii or Talaromyces or Yersinia pestis or Francisella tularensis or Klebsiella pneumoniae or Mycoplasma).ti,ab.(164201) |
| 43 | (Q fever or salmonellosis or salmonella infection* or melioidosis or brucellosis or listeria infection* or listeriosis or leptospira* infection* or leptospirosis or rickettsia* infection* or rickettsios* or enteric fever or typhoid fever or paratyphoid fever or plague or anaplasmosis or ehrlichiosis or cat scratch fever or cat scratch disease or trench fever or Carrion* disease or borreliosis or lyme or bartonellosis or relapsing fever or typhus or blastomycosis or cryptococcal or coccidioidomycosis or valley fever or histoplasmosis or penicilliosis or tularaemia or tularemia).ti,ab.(72205) |
| 44 | ((gram-positive adj3 (bacteria* or infection*)) or (gram-negative adj3 (bacteria* or infection*))).ti,ab.(16651) |
| 45 | Cerebrospinal Fluid/ or (cerebrospinal fluid or csf).ti,ab.(19853) |
| 46 | exp bacterial diseases/ not bacteraemia/(305606) |
| 47 | exp bacteria/(558929) |
| 48 | or/10-47(808894) |
| 49 | man/(1811686) |
| 50 | exp animals/(2660059) |
| 51 | 50 not (49 and 50)(848373) |
| 52 | exp China/ or China.ti,ab.(135576) |
| 53 | (Anhui or Fujian or Guizhou or Hainan or Henan or Hubei or Jiangsu or Yunnan or Zhejiang or Guangxi or Tibet or Xizang or Chongqing).ti,ab.(16104) |
| 54 | 52 or 53(136007) |
| 55 | 6 and 48 and 54(5780) |
| 56 | 9 and 54(4615) |
| 57 | 55 or 56(10153) |
| 58 | 57 not 51(9446) |
| 59 | limit 58 to ((chinese or english or french or portuguese or spanish) and yr="1980-Current")(8759) |

| **3. China: Embase <1974 to 2016 May 03> (Searched May 04, 2016)** | |
| --- | --- |
| 1 | exp Anti-Bacterial Agents/dt, pd, tu, th, ut [Drug Therapy, Pharmacology, Therapeutic Use, Therapy, Utilization](1019791) |
| 2 | ext Bacteremia/ or (bacteremi* or bacteraemi* or septicemi* or septicaemi*).ti,ab.(52725) |
| 3 | exp Sepsis/ or ("blood stream infection*" or "bloodstream infection*" or "blood stream pathogen*" or "bloodstream pathogen*" or (("blood borne" or bloodborne or blood-borne) adj (infection* or pathogen*))).ti,ab.(207217) |
| 4 | exp Fever/ or (febrile or fever* or pyrexia or pyrexic or pyrexial or pyrexiae or hyperthermi* or (raised adj2 temperature) or (high adj 2 temperature) or (elevated adj2 temperature)).ti,ab.(332817) |
| 5 | or/1-3(1192379) |
| 6 | (babesi* or leishmania* or chagas or trypanosom* or "sleeping sickness").ti,ab.(69993) |
| 7 | Babesia microti/ (740) |
| 8 | exp Trypanosoma/ (27499) |
| 9 | exp Trypanosomiasis/ not Trypanosomiasis, Bovine/ (22373) |
| 10 | exp Leishmania/ (22657) |
| 11 | exp Leishmaniasis/ (24585) |
| 12 | Influenza A Virus, H1N1 Subtype/ or Influenza A virus/ or Influenza a virus.ti,ab.(9658) |
| 13 | exp Arbovirus infections/ or Arboviruses/ or exp Encephalitis viruses, Japanese/ or Encephalitis virus, Japanese/ or Encephalitis virus, Murray Valley/ or "Encephalitis virus, St. Louis"/ or West Nile virus/ or Encephalitis viruses, tick-borne/ or Yellow fever virus/ or (Japanese Encephalitis or Japanese B Encephalitis or Phlebotomus fever or Rift Valley fever or Yellow fever or ((California or "St Louis" or Tick-borne) adj Encephalitis) or Kyasanur Forest disease).ti,ab.(971118) |
| 14 | Chikungunya virus/ or Chikungunya.ti,ab. (3120) |
| 15 | exp Epstein-Barr Virus Infections/ or Epstein-Barr.ti,ab. (44735) |
| 16 | exp Dengue/ or Dengue Virus/ or Dengue.ti,ab. (20641) |
| 17 | Respiratory Syncytial Virus, Human/ or Respiratory Syncytial Virus Infections/ or (Respiratory Syncytial Virus or RSV).ti,ab.(17212) |
| 18 | Measles/ or Measles virus/ or Measles.ti,ab.(29178) |
| 19 | Hantavirus/ or Hantaan Virus/ or Puumala Virus/ or Seoul Virus/ or Sin Nombre Virus/ or Hantavirus Infections/ or Hantavirus Pulmonary Syndrome/ or Hemorrhagic Fever with Renal Syndrome/ or (Hantavirus or Hantaan or Puumula or Seoul virus or Sin Nombre virus or Hemorrhagic fever or Haemorrhagic fever).ti,ab.(12330) |
| 20 | Nipah Virus/ or Nipah Virus.ti,ab. (910) |
| 21 | Coxsackievirus Infections/ or (Coxsackie or Coxsackievirus*).ti,ab.(9848) |
| 22 | Cytomegalovirus Infections/ or Cytomegalovirus/ or Cytomegalovirus*.ti,ab.(55830) |
| 23 | Hepatitis/ or Hepatitis, Viral, human/ or Hepatitis a/ or Hepatitis b/ or Hepatitis b, chronic/ or Hepatitis c/ or Hepatitis c, chronic/ or Hepatitis e/ or Hepatitis b virus/ or Hepacivirus/ or Hepatitis e virus/ or Hepatitis a virus/ or Hepatitis a virus, human/ or (Hepatitis adj (a or b or c or e)).ti,ab.(266064) |
| 24 | (Coxiella burnetii or Salmonella typhi or Salmonella paratyphi or Burkholderia pseudomallei or Pseudomonas pseudomallei or Brucella or Escherichia coli or E-coli or E coli or Citrobacter freundii or Listeria monocytogenes or (Leptospira adj (kmetyi or interogans or weilii or parva)) or Rickettsia or Orientia tsutsugamushi or mycobacterium tuberculosis or h1n1 or Human Herpesvirus 4 or Human Herpes virus 4 or Ehrlichia or Anaplasma or Bartonella or Borrelia or Neorickettsia or Sennetsu or Blastomyces or Cryptococcus or Coccidioides or Histoplasma or Penicillium marneffii or Talaromyces or Yersinia pestis or Francisella tularensis or Klebsiella pneumoniae or Mycoplasma).ti,ab.(447194) |
| 25 | (Q fever or salmonellosis or salmonella infection* or melioidosis or brucellosis or listeria infection* or listeriosis or leptospira* infection* or leptospirosis or rickettsia* infection* or rickettsios* or enteric fever or typhoid fever or paratyphoid fever or plague or anaplasmosis or ehrlichiosis or cat scratch fever or cat scratch disease or trench fever or Carrion* disease or borreliosis or lyme or bartonellosis or relapsing fever or typhus or blastomycosis or cryptococcal or coccidioidomycosis or valley fever or histoplasmosis or penicilliosis or tularaemia or tularemia).ti,ab.(89146) |
| 26 | Blood-Borne Pathogens/ (1745) |
| 27 | ((gram-positive adj3 (bacteria* or infection*)) or (gram-negative adj3 (bacteria* or infection*))).ti,ab. (52668) |
| 28 | Cerebrospinal Fluid/ or (cerebrospinal fluid or csf).ti,ab. (186637) |
| 29 | exp Bacterial Infections/ not exp Bacteremia/ (798756) |
| 30 | exp Bacteria/ (1410980) |
| 31 | or/6-30(3211259) |
| 32 | exp Animals/ (21613093) |
| 33 | Humans/ (11956394) |
| 34 | 32 not (32 and 33)(9659888) |
| 35 | exp China/ or China.ti,ab.(167847) |
| 36 | (Anhui or Fujian or Guizhou or Hainan or Henan or Hubei or Jiangsu or Yunnan or Zhejiang or Guangxi or Tibet or Xizang or Chongqing).ti,ab.(22326) |
| 37 | Anhui/ or Fujian/ or Guizhou/ or Hainan/ or Henan/ or Hubei/ or Jiangsu/ or Yunnan/ or Zhejiang/ or Guangxi/ or Tibet/ or Xizang/ or Chongqing/(111612) |
| 38 | or/35-37(174896) |
| 39 | 5 and 31 and 38(3504) |
| 40 | 4 and 38(3337) |
| 41 | 39 or 40(6349) |
| 42 | 41 not 34(2916) |
| 43 | limit 42 to (chinese or english or french or portuguese or spanish)(2844) |
| 44 | limit 43 to yr="1980-Current"(2826) |
| 45 | limit 44 to exclude medline journals(291) |

4. CNKI was searched on May 12, 2016

Remarks:

- Professional Search
- Timespan: Auto (exclude reports published before 1980 manually @ EndNote)
- Language: All Languages
- Search topic: Medicine & Public Health - Journals
- Search Terms used: SU=Topic (including title + abstract)
  Due to word limit (500 words), multiple steps
- General + GA -> Related Disease (by using “Search in result” function)
- Fever + GA
- (a+b) Duplicate reports – EndNote
- Did not exclude “Not (SU=Animals)” in the search, as it will exclude those records with “Animals” term in title or abstract, which may exclude some relevant records eg. Plague, swine flu

*The exact search terms (in Chinese) adopted are removed from this supplemental file upon request from the handling editor. The corresponding authors may be contacted for specific search details adopted for this database*.

5. China: Web of Science – Chinese Science Citation Database (Searched May 12, 2016)

Remarks:

- Advanced Search
- Timespan: Set “All years” (from 1989-2016 only)
- Language: All Languages
- Search Terms used: TS=Topic
  Searches for topic terms in the following fields within a record.
- Title
- Abstract
- Author Keywords

*The exact search terms (in Chinese) adopted are removed from this supplemental file upon request from the handling editor. The corresponding authors may be contacted for specific search details adopted for this database*.

6. China: WanFang Med Online- (Searched May 16-30, 2016)

Remarks:

1. Advanced Search only allow simple combinations of the search words – up to five combinations
2. Timespan: All years (from 1998 – 2016 only)
3. Language: All Languages
4. Search topic: Journals
5. Search Terms used: Title and Abstract
6. Duplicate reports – EndNote
7. Did not exclude “Title=Animals or abstract=Animals” in the search, as it may exclude some relevant records eg. Plague, swine flu

*The exact search terms (in Chinese) adopted are removed from this supplemental file upon request from the handling editor. The corresponding authors may be contacted for specific search details adopted for this database*.
