# Supplementary material for: Non-malarial febrile illness: a systematic review of published aetiological studies and case reports from China, 1980–2015
Source: BMC Infect Dis. 2024 Aug 20;24:843. doi: 10.1186/s12879-024-09542-3 (PMC11334328; doi:10.1186/s12879-024-09542-3)

**Supplemental file 3: Additional figures**

**Non-malarial febrile illness: a systematic review of published aetiological studies and case reports from China, 1980-2015**

Dennis K. M. Ip^*^, Yvonne Y. Ng, Yat H. Tam, Nigel V. Thomas, Prabin Dahal, Kasia Stepniewska, Paul N. Newton, Philippe J. Guérin, Heidi Hopkins^*^

*[dkmip@hku.hk](mailto:dkmip@hku.hk)

*[Heidi.Hopkins@lshtm.ac.uk](mailto:Heidi.Hopkins@lshtm.ac.uk)

**S Figure 1: Most commonly reported bacterial pathogens**


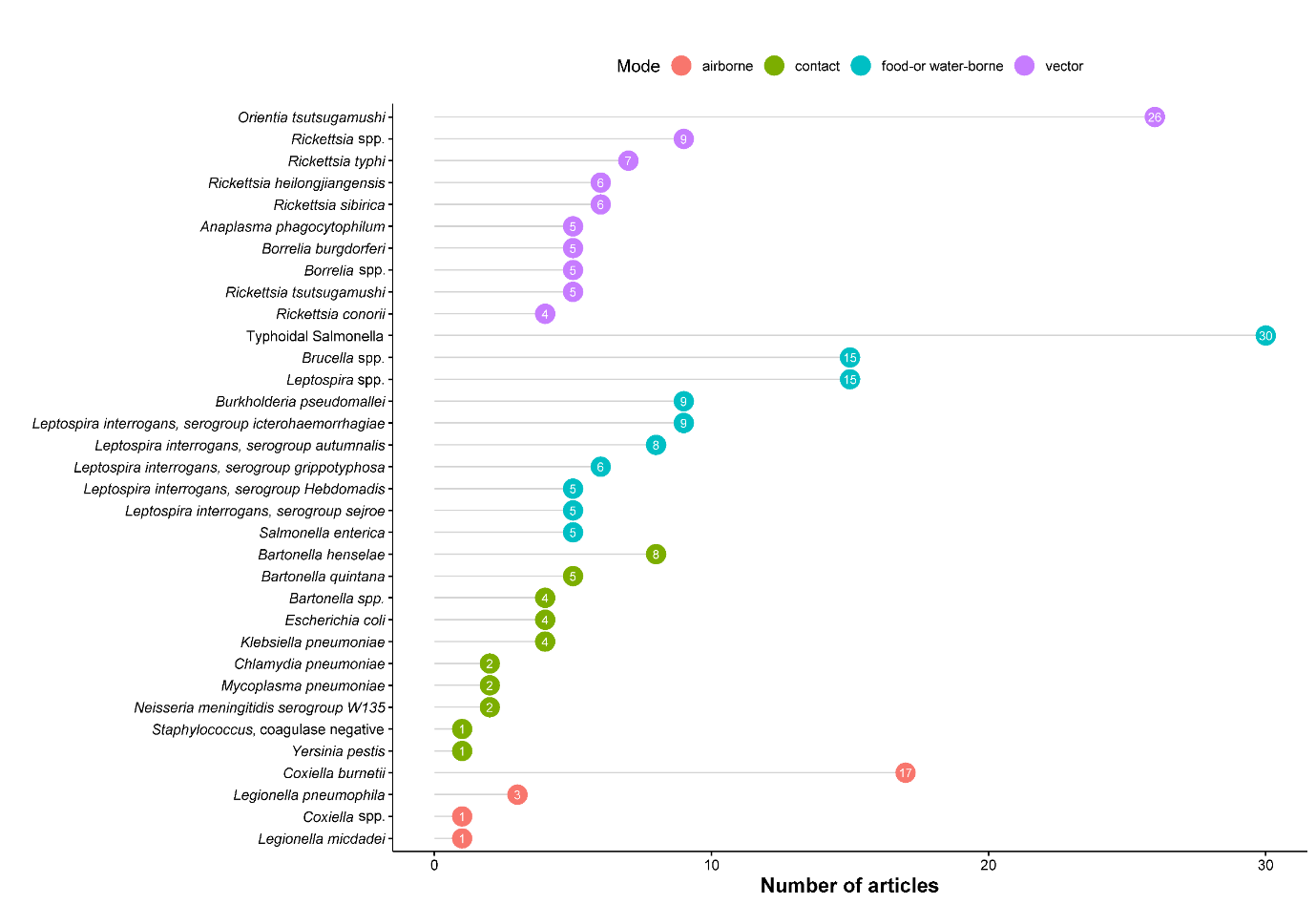


**S Figure 2: Most commonly viruses**


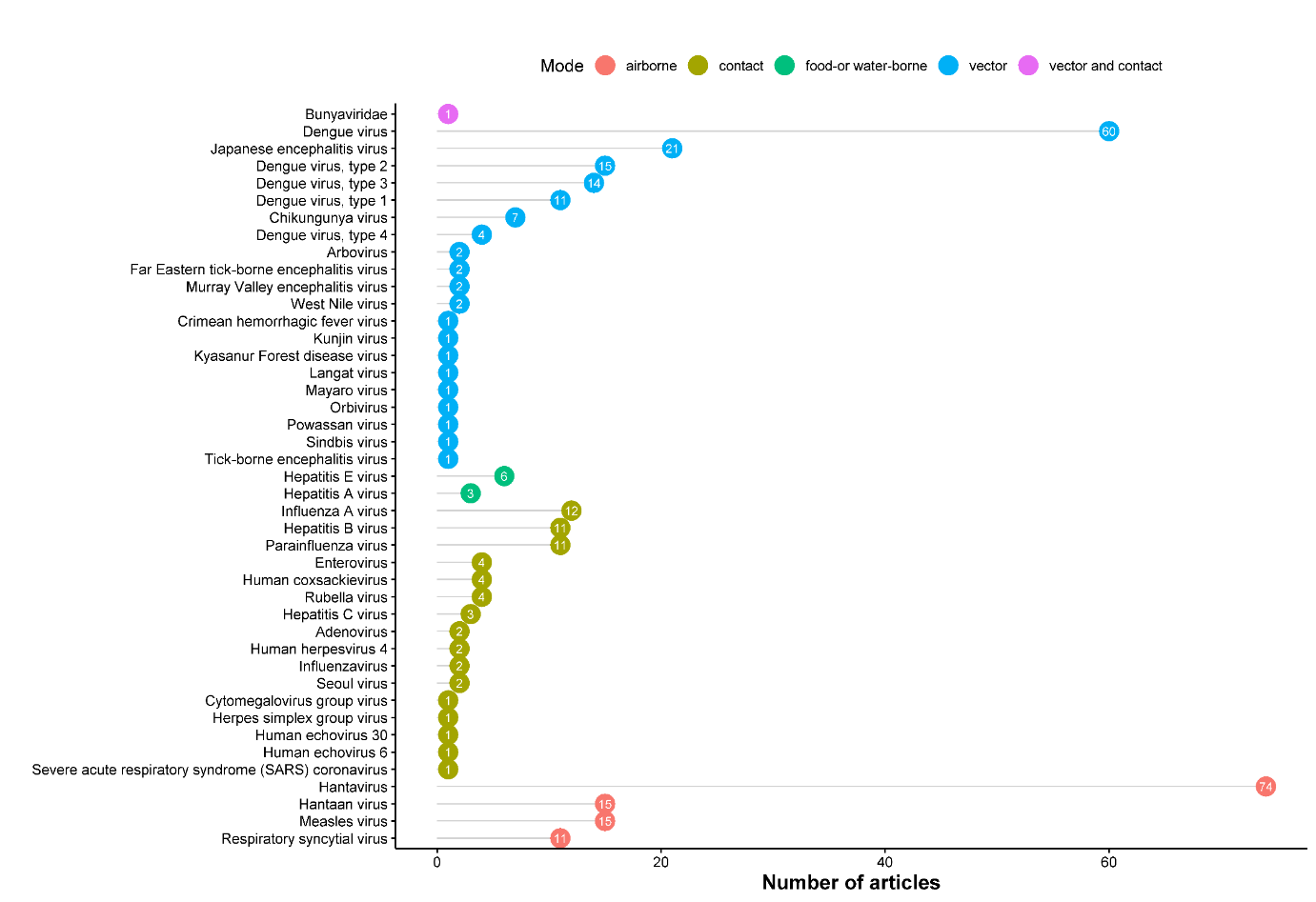


**S Figure 3: All reported parasites and fungus**


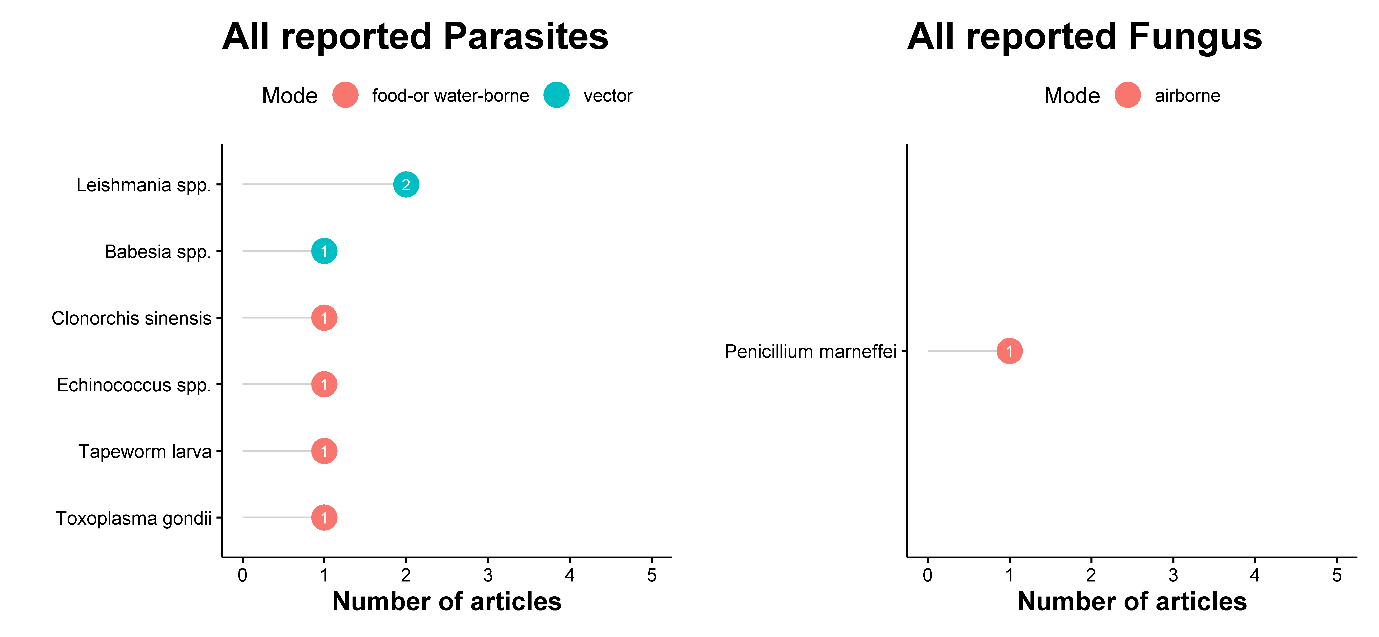


**Legend:** One article reported fungus without stating the genus and species (not shown on this plot)

**S Figure 4: Pathogens reported in Anhui**


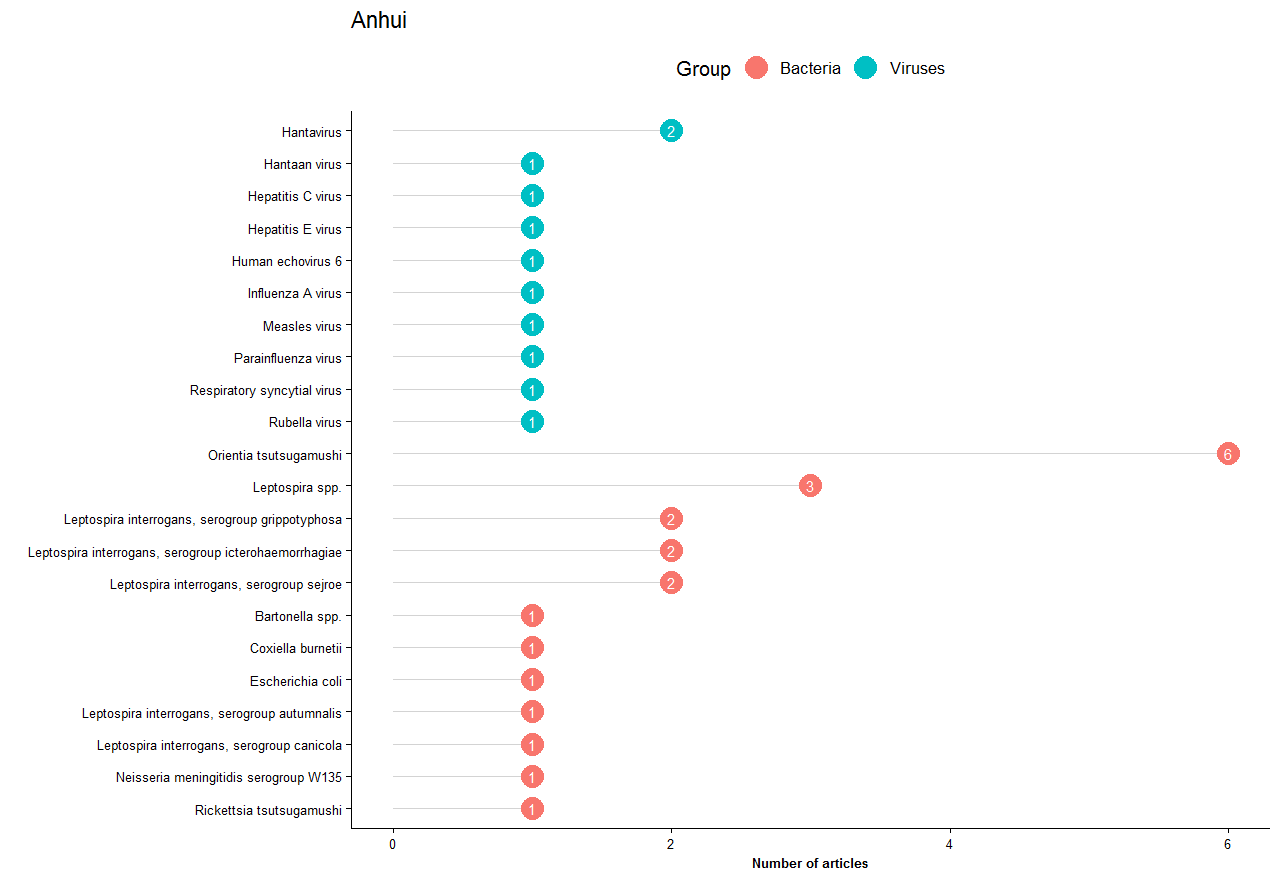


**S Figure 5: Pathogens reported in Chongqing**


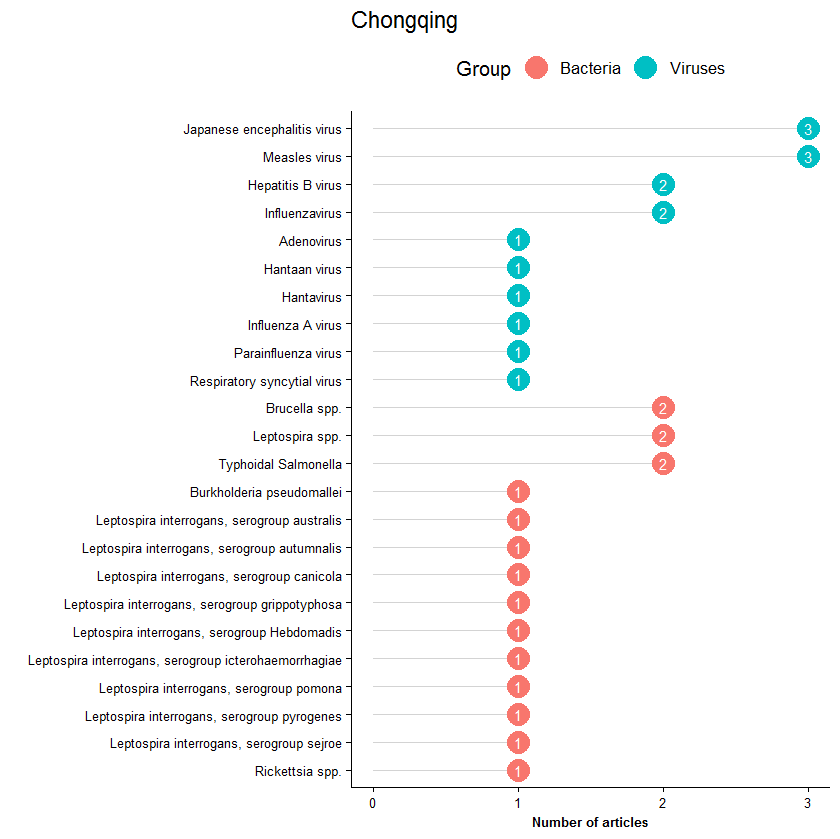


**S Figure 6: Pathogens reported in Fujian**


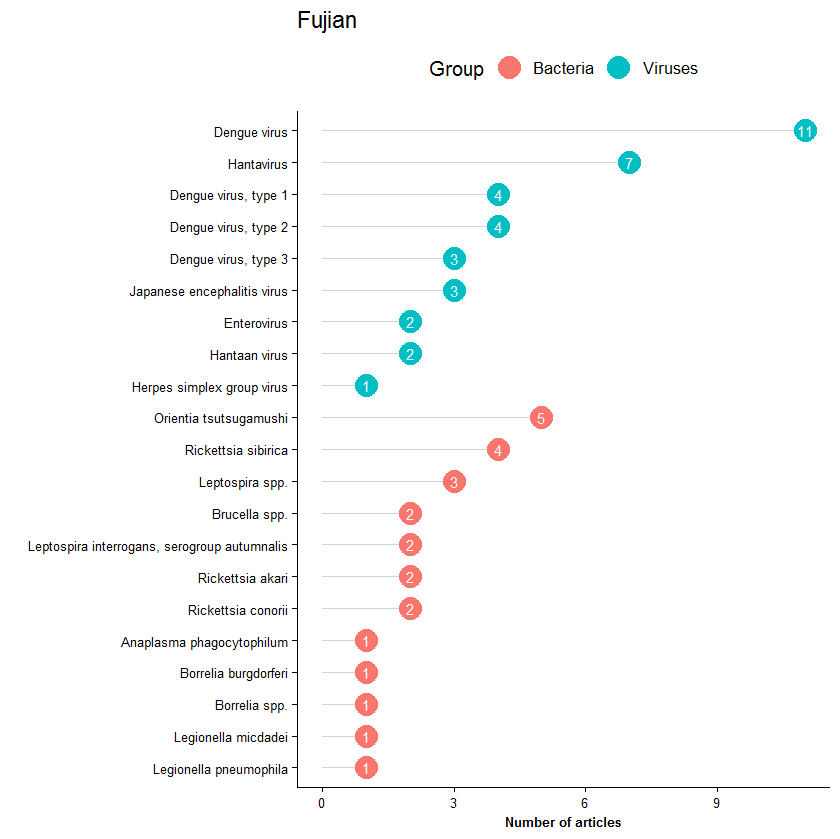


**S Figure 7: Pathogens reported in Guangxi**


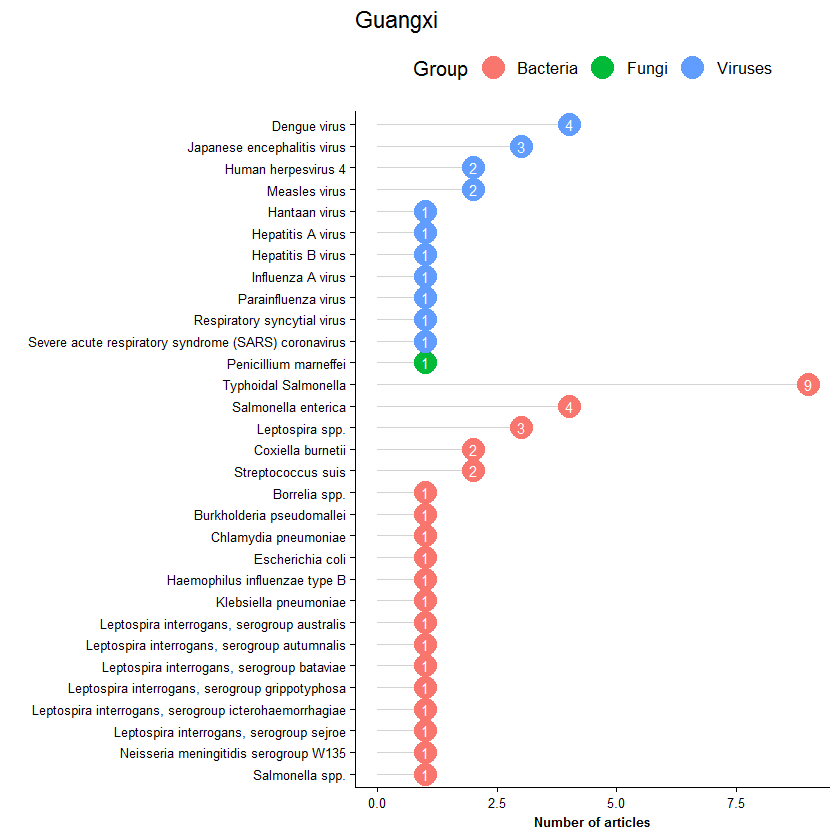


**S Figure 8: Pathogens reported in Guizhou**


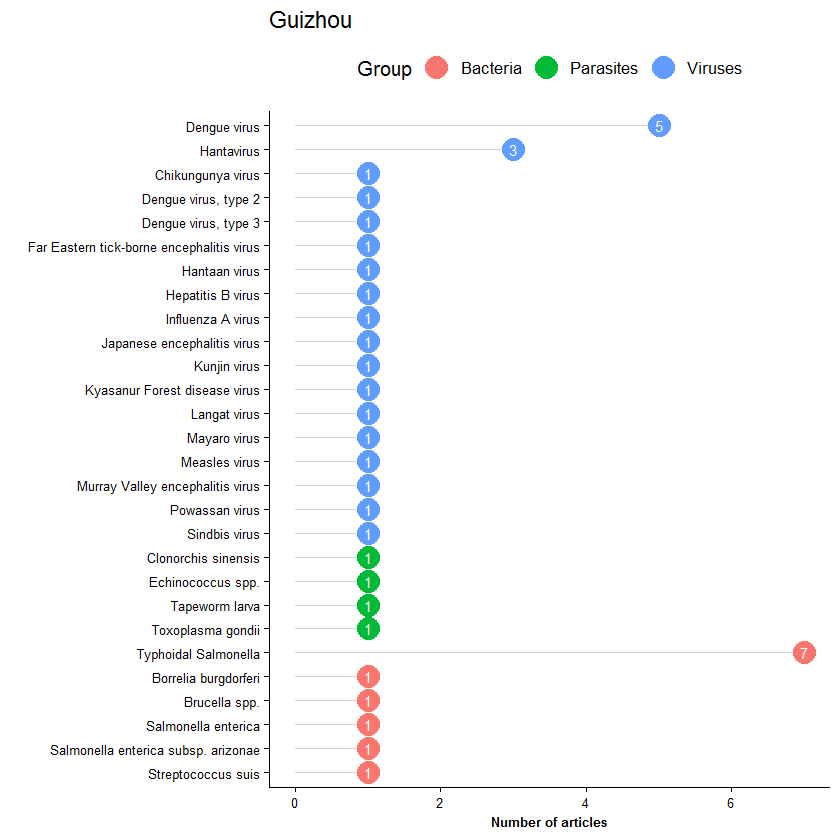


**S Figure 9: Pathogens reported in Hainan**


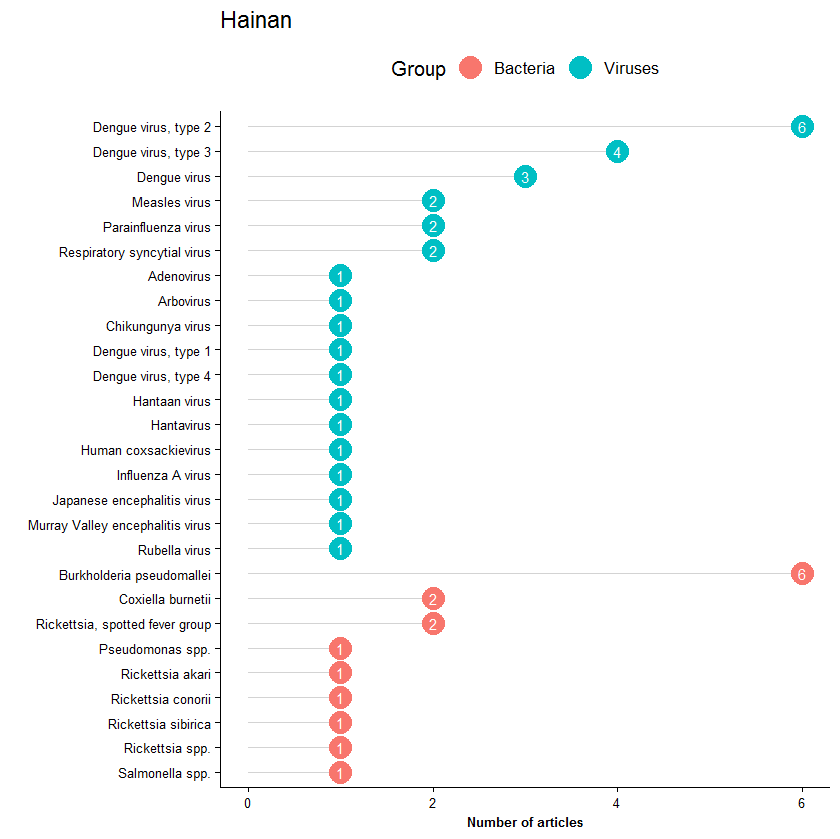


**S Figure 10: Pathogens reported in Henan**


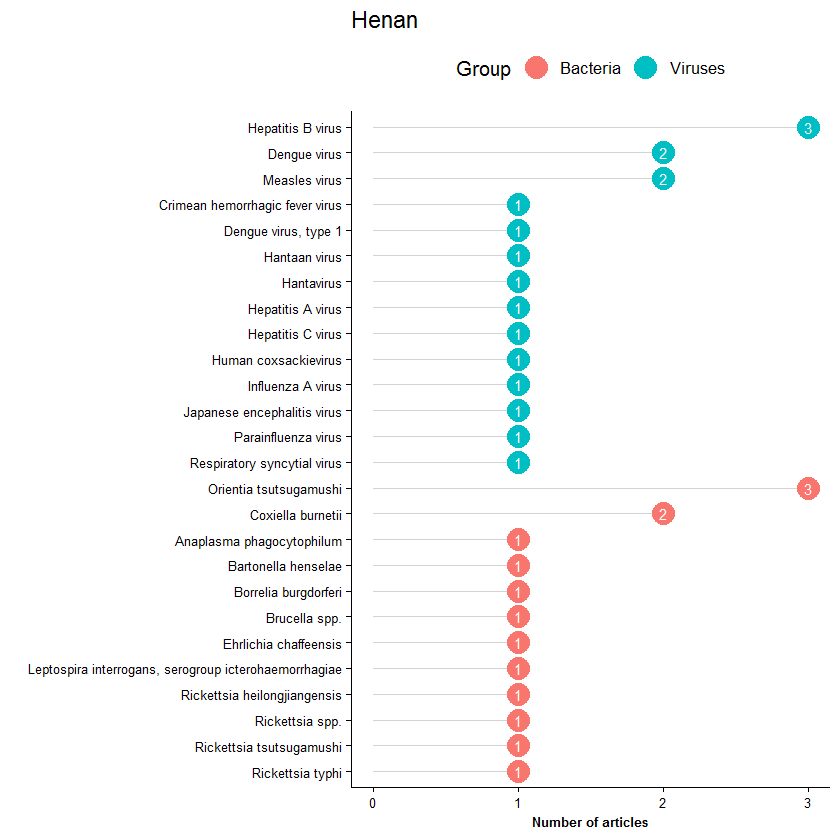


**S Figure 11: Pathogens reported in Hubei**


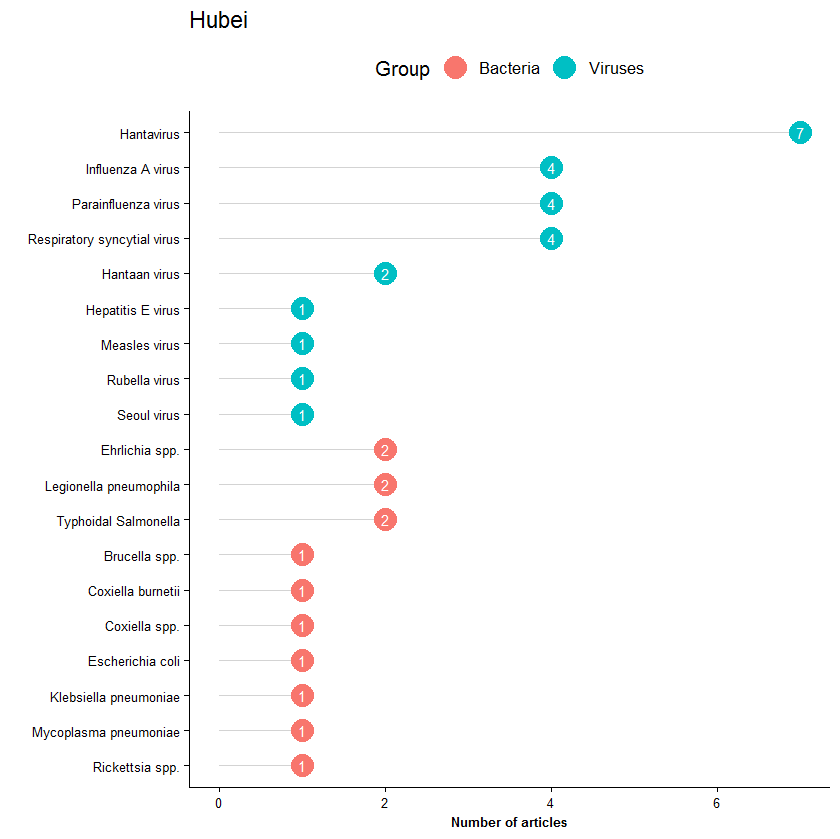


**S Figure 12: Pathogens reported in Jiangsu**


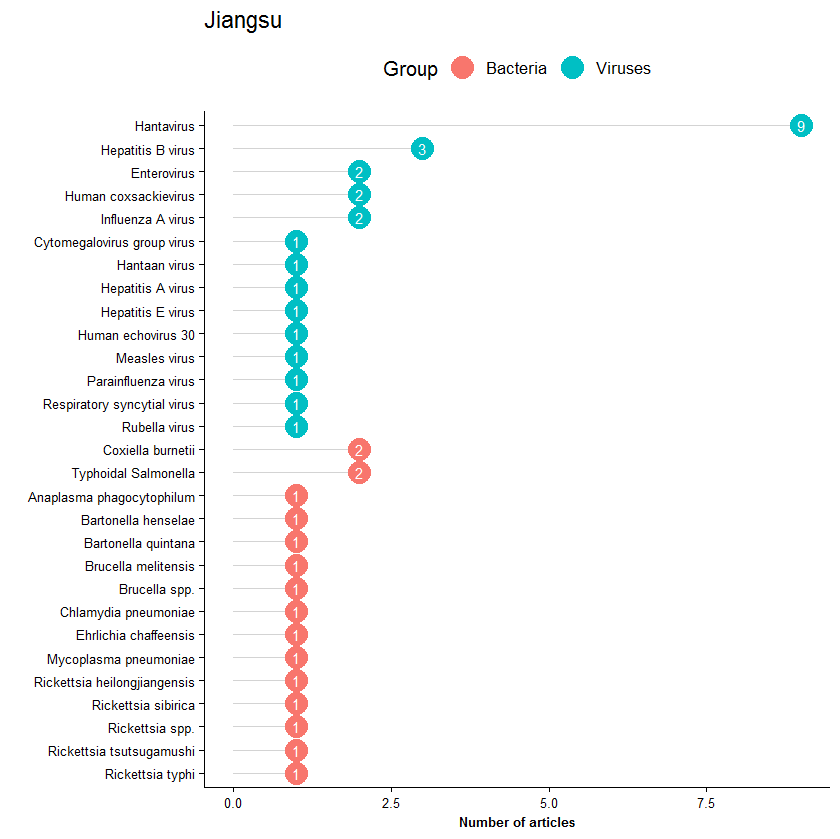


**S Figure 13: Pathogens reported in Tibet**


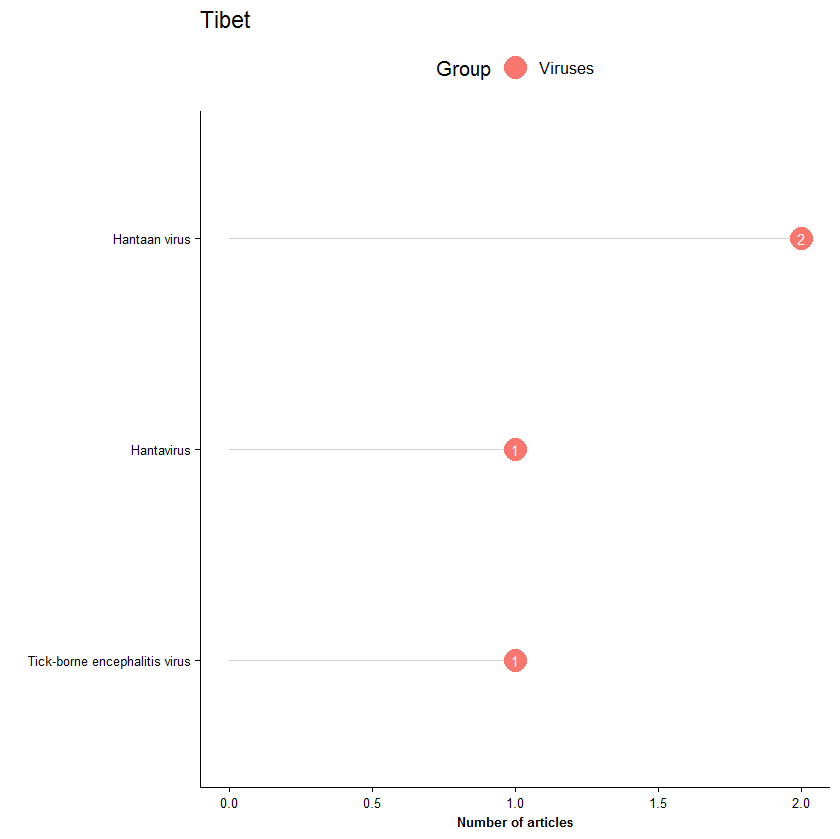


**S Figure 14: Pathogens reported in Yunnan**


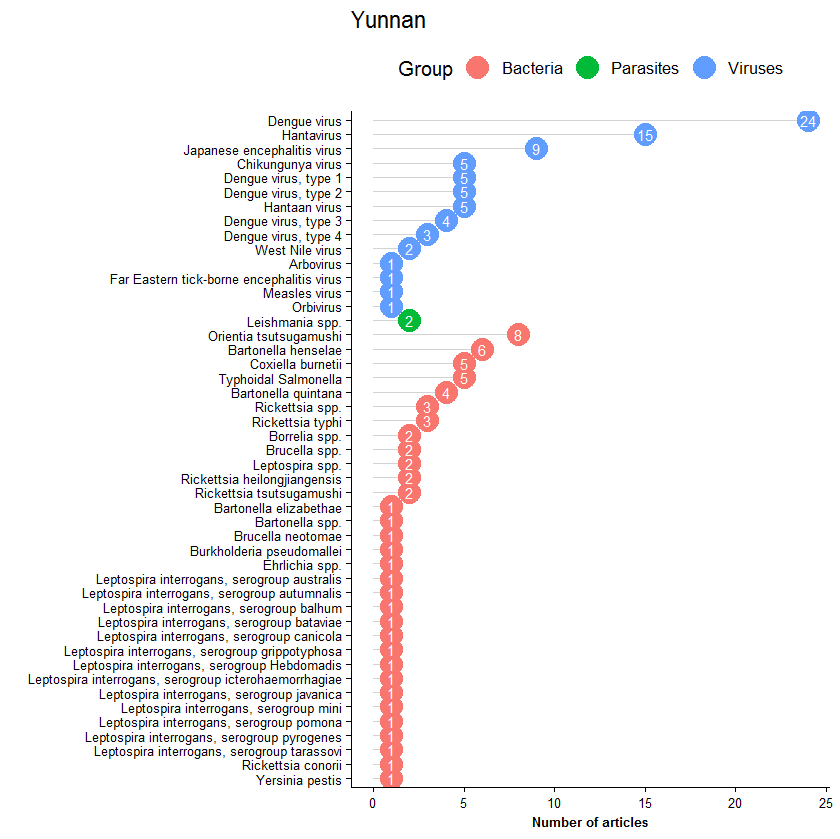


**S Figure 15: Pathogens reported in Zhejiang**


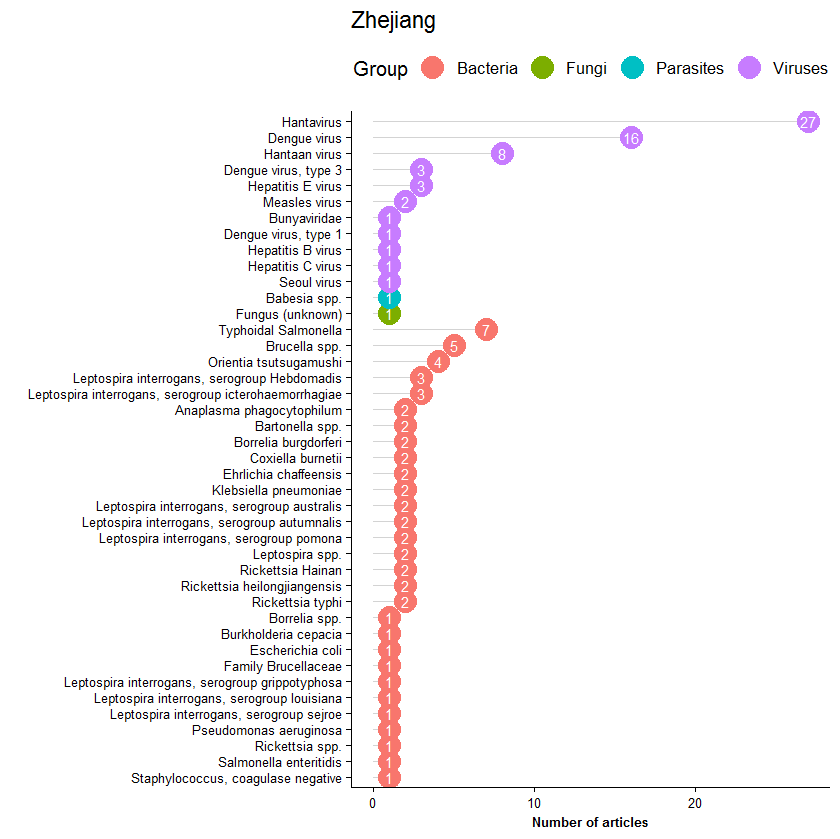

Supplement: Supplementary file 2 — Supplementary Material 2 [file 12879_2024_9542_MOESM2_ESM.docx]
